# Cerebellar organoids model cell type-specific *FOXP2* expression during human cerebellar development

**DOI:** 10.1242/dmm.052290

**Published:** 2025-11-14

**Authors:** Elizabeth J. Apsley, Joey Riepsaame, Yin Chun Cheng, Sally A. Cowley, Esther B. E. Becker

**Affiliations:** ^1^ Nuffield Department of Clinical Neurosciences, University of Oxford, Oxford OX3 9DU, UK; ^2^ Kavli Institute of Nanoscience Discovery, University of Oxford, Oxford OX1 3QU, UK; ^3^ Genome Engineering Oxford, Sir William Dunn School of Pathology, University of Oxford, Oxford OX1 3RE, UK; ^4^ James and Lillian Martin Centre for Stem Cell Research, Sir William Dunn School of Pathology, University of Oxford, Oxford OX1 3RE, UK

**Keywords:** Cerebellum, Purkinje cells, Cerebellar nuclei, Forkhead box, iPSC, Neurodevelopmental disorder

## Abstract

Human cerebellar development is unique and cannot be fully replicated in animal models. Although human stem cell-derived cerebellar organoid models are increasingly being applied to model cerebellar diseases, their potential to provide insight into normal human cerebellar development remains underexplored. Here, we used CRISPR-based gene editing in cerebellar organoids as an approach for modelling specific features of early human cerebellar development. Forkhead box protein P2 (FOXP2) is a transcription factor associated with speech and language development that is highly expressed in the developing brain. However, little attention has been directed to the study of FOXP2 in the early developing cerebellum. We generated a fluorescent FOXP2 reporter line in human induced pluripotent stem cells to enable the characterisation of FOXP2-expressing cells during cerebellar organoid differentiation. Through transcriptomic profiling of FOXP2 reporter cerebellar organoids and cross-referencing with existing cerebellar datasets, we describe the expression and identify potential downstream targets of FOXP2 in the early developing human cerebellum. Our results highlight expression of *FOXP2* in early human Purkinje cells and cerebellar nuclei neurons, and the vulnerability of these cell populations to neurodevelopmental disorders.

## Teaser

**Summary:** Using a cerebellar organoid model, we characterise expression of FOXP2 in the early human developing cerebellum, with implications for vulnerability to neurodevelopmental disorders.

## INTRODUCTION

Human cerebellar development is a highly orchestrated process that is vulnerable to a broad spectrum of neurodevelopmental disorders (NDDs) (Haldipur et al., 2022; ten Donkelaar and Lammens, 2009). Recent studies have highlighted important differences between mouse and human cerebellar development, including in the Purkinje cell lineage (Haldipur et al., 2019, 2022; Sepp et al., 2024). Human cerebellar development is highly protracted, and progenitor zones in the human developing cerebellum that give rise to the glutamatergic and GABAergic neuronal populations are more complex (Haldipur et al., 2019, 2022). In addition, the developing human cerebellum exhibits significantly higher abundance of Purkinje cells and a shift in the relative proportions of embryonic Purkinje cell subtypes compared to those in mouse (Sepp et al., 2024). These studies underscore a need for human-centric models to gain insights into mechanisms underlying early human cerebellar development.

The differentiation of human induced pluripotent stem cells (iPSCs) into cerebellar neurons and organoids has been shown to recapitulate early stages of cerebellar development (Atamian et al., 2024; Muguruma et al., 2015; Silva et al., 2020; Watson et al., 2018) and thus provides a promising human-specific model to probe the specification and maturation of cerebellar neurons. Single-cell RNA-sequencing (scRNAseq) studies have profiled the cell types present in differentiated cerebellar organoids, including populations with characteristics of granule cells and Purkinje cells, the major populations of neurons in the cerebellum (Atamian et al., 2024; Chen et al., 2023; Nayler et al., 2021). Moreover, following prolonged culture, iPSC-derived cerebellar organoids and plated neurons have been shown to develop spontaneous neuronal activity (Atamian et al., 2024; Chen et al., 2023; Silva et al., 2020). Following the establishment of robust methods, to date, iPSC-derived cerebellar models have been used to study the mechanisms of cerebellar diseases such as ataxia, medulloblastoma and pontocerebellar hypoplasia (Ballabio et al., 2020; Ishida et al., 2016; Kagermeier et al., 2024; van Essen et al., 2024). However, cerebellar organoid models have not yet been applied to investigate aspects of normal human cerebellar development.

In this study, we used iPSC-derived cerebellar organoids to investigate the development and properties of cells expressing the speech and language-associated gene *FOXP2* in the early human cerebellum. This gene encodes Forkhead box protein P2 (FOXP2), a transcription factor of the evolutionarily conserved FOXP subfamily. Over two decades ago, a variant in *FOXP2* was identified as the monogenic cause of a severe speech and language disorder in a large multigenerational family (Lai et al., 2001). However, despite considerable work, there is still relatively little understanding of the molecular mechanisms that underlie the function of FOXP2 and how this contributes to language development (den Hoed et al., 2021). During development, *FOXP2* is expressed across several brain regions including the cerebellum (Co et al., 2020; Ferland et al., 2003; Fujita and Sugihara, 2012). Interestingly, cerebellar alterations have been identified in neuroimaging studies of individuals affected by *FOXP2*-related disorders and in genetic models of *FOXP2* disruption (Argyropoulos et al., 2019; Co et al., 2020; Liégeois et al., 2011; Watkins et al., 2002). Mice with homozygous disruption of *Foxp2* showed decreased size and foliation of the cerebellum, whereas heterozygous mice showed little or no gross cerebellar changes (French et al., 2007; Fujita et al., 2008; Groszer et al., 2008; Shu et al., 2005). Cerebellar-specific knockdown of *Foxp2* in mice during embryonic development demonstrated the contribution of FOXP2 action in the cerebellum to normal brain function by reproducing some of the phenotypes of global mutations, including impaired Purkinje cell dendritic development, gross motor deficits and altered vocalisation (Usui et al., 2018). In contrast, postnatal Purkinje cell knockout of *Foxp2* caused impaired performance only in more skilled motor tasks (French et al., 2019). These differences in timing of *Foxp2* disruption and the severity of the resulting phenotype suggest that *Foxp2* has a particularly important role in newly differentiated Purkinje cells. However, studies of *Foxp2* disruption have largely focused on alterations in Purkinje cell dendritic development, without examining earlier stages of Purkinje cell differentiation or characterising other cerebellar cell types in detail.

To better understand the role of *FOXP2* in the specification and maturation of Purkinje cells in humans, we employed a human-specific cerebellar organoid model. We first showed that FOXP family genes *FOXP1*, *FOXP2* and *FOXP4* were robustly induced during organoid differentiation. We then generated a *FOXP2* reporter line and performed detailed characterisation of *FOXP2*-expressing cells in human cerebellar organoids. Transcriptomic analysis of these cells identified features of Purkinje cells and cerebellar nuclei (CN) neurons, corroborated by cross-referencing with human cerebellar datasets. We found that *FOXP2*^+^ cerebellar neurons expressed a high number of genes associated with specific diseases including autism spectrum disorder (ASD), suggesting that these cells in the developing human cerebellum are specifically vulnerable in NDDs. Together, our work demonstrates the value of cerebellar organoids to model early human developmental processes and gain insight into distinct disease mechanisms.

## RESULTS

### FOXP genes show distinct expression patterns in developing Purkinje cells

**Fig. 1.**
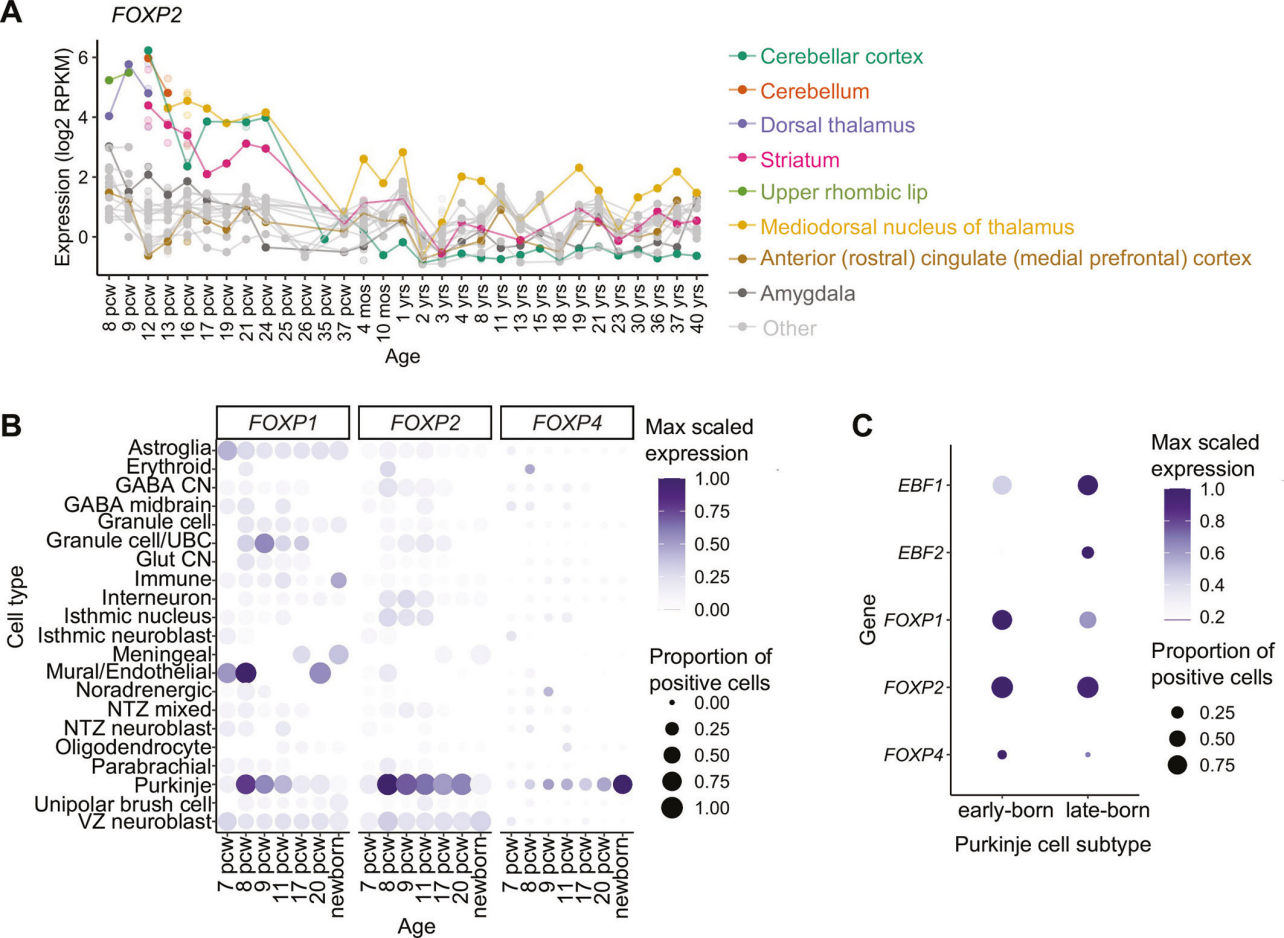
***FOXP2* is expressed highly in developing human Purkinje cells.** (A) *FOXP2* is highly expressed in the cerebellum during human brain development. Bulk RNA-sequencing data from BrainSpan. Regions with highest expression are depicted in colour, with other brain regions shown in grey. Where multiple samples were present for a timepoint, the mean value is a solid circle. Individual samples are represented by open circles. mos, months after birth; pcw, post-conception weeks; RPKM, reads per kilobase of transcript per million mapped reads; yrs, years after birth. (B) Expression of FOXP genes in the developing human cerebellum plotted across timepoints and cell types based on published single-nucleus RNA-sequencing (snRNAseq) data (Sepp et al., 2024). Point size indicates the proportion of expressing cells in each cluster, and the colour scale shows average expression levels. Samples with <10 cells were excluded. (C) FOXP genes show differential expression across Purkinje cell subtypes, with *FOXP1* marking early-born Purkinje cells [false discovery rate (FDR)=4.58×10^−302^, log2 fold change (log2FC)=0.613]. *FOXP2* (FDR=1.82×10^−22^, log2FC=0.158) and *FOXP4* (FDR=8.51×10^−20^, log2FC=0.0436) are slightly enriched in the early-born Purkinje cell population, in contrast to late-born markers *EBF1* (FDR=2.16×10^−304^, log2FC=0.725) and *EBF2* (FDR=5.40×10^−165^, log2FC=0.302). Analysis based on snRNAseq data from Sepp et al. (2024).

We first examined the expression of *FOXP2* in the developing human cerebellum using published transcriptomic datasets. Plotting *FOXP2* mRNA levels across human brain development, using the BrainSpan bulk RNA-sequencing dataset, confirmed particularly high *FOXP2* expression in the cerebellum, striatum and dorsal thalamus in the first and second trimester (Fig. 1A). By birth, *FOXP2* expression in the cerebellar cortex decreased substantially (Fig. 1A). This time period coincides with a shift in cell type proportions in the cerebellum due to a massive expansion of granule cells and therefore relative decrease in the Purkinje cell population (Aldinger et al., 2021; Sepp et al., 2024). Within the cerebellum, single-nucleus RNA-sequencing (snRNAseq) data from the prenatal human cerebellum confirmed strong *FOXP2* expression in Purkinje cells from 8 post conception weeks (PCW) (Fig. 1B) (Sepp et al., 2024). *FOXP2* expression remained high in Purkinje cells up to 20 PCW, but was decreased at birth consistent with the bulk RNA-sequencing data (Fig. 1A,B) (Sepp et al., 2024). The low number of Purkinje cells captured in postnatal samples limited characterisation of expression at later timepoints.

Dimerisation of FOXP2 is essential for DNA binding and transcriptional regulation (Li et al., 2004). FOXP2 can form both homodimers or heterodimers with the closely related FOXP1 or FOXP4, with combinations differentially regulating target genes (Li et al., 2004; Sin et al., 2015). Therefore, the combinations of other FOXP proteins co-expressed with FOXP2 are significant to its function. To consider the possible FOXP protein interactions in the developing human cerebellum, we also analysed the expression of *FOXP1* and *FOXP4* in transcriptomic data of the developing human cerebellum. Both *FOXP1* and *FOXP4* were enriched in the Purkinje cell cluster and thus overlapping with *FOXP2*, although with different trajectories (Fig. 1B). Similarly to *FOXP2* expression, *FOXP1* expression peaked at 8 PCW and decreased over time. However, compared to *FOXP2* expression, *FOXP1* expression showed an earlier decline, with Purkinje cell expression dropping by 17 PCW (Fig. 1B). In contrast, *FOXP4* expression increased at later stages of prenatal development, with highest expression occurring in the newborn cerebellum (Fig. 1B). Similar trends in Foxp gene expression were found in the developing mouse cerebellum (Fig. S1A). *Foxp*2 and *Foxp4* were most highly expressed in Purkinje cells, with levels of *Foxp2* peaking in development and levels of *Foxp4* increasing later in adult Purkinje cells, as previously described (Fig. S1A) (Ferland et al., 2003; Lai et al., 2003; Tam et al., 2011). Expression of *Foxp1* in mouse Purkinje cells was relatively low overall but, as in human, peaked early in embryonic development (Fig. S1A).

Purkinje cells are known to be a heterogeneous population and can be grouped into subtypes on the basis of gene expression and location from early embryonic development (Apsley and Becker, 2022; Fujita et al., 2012; Hashimoto and Mikoshiba, 2003). In mouse, the differential expression of *Foxp1* and *Foxp2* has been suggested to contribute to defining embryonic Purkinje cell subpopulations, with multiple studies defining a subtype exhibiting high *Foxp1* expression (Sepp et al., 2024; Wizeman et al., 2019). To investigate whether this also applies in human development, we compared FOXP gene expression across developmental human Purkinje cell subtypes. Human embryonic Purkinje cell subtypes have been defined by timing (early- and late-born) and by gene expression, including the low or high expression of *EBF1* and *EBF2* genes (Sepp et al., 2024). *FOXP2* showed expression across both subtypes, although we found a small, but significant, enrichment in the early-born, *EBF1/2*-low subtype [false discovery rate (FDR)=1.8×10^−22^, log2 fold change (log2FC)=0.158]. *FOXP1* and *FOXP4* expression was strongly enriched in the early-born, EBF1/2-low Purkinje cell subtype, and little expression was found in the late-born, EBF1/2-high Purkinje cells (*FOXP1* FDR=4.58×10^−302^, log2FC=0.613; *FOXP4* FDR=8.51×10^−20^, log2FC=0.0436) (Fig. 1C). We also observed similar expression of Foxp genes across mouse Purkinje cell subtypes. Four developmental mouse Purkinje cell subtypes have been described by timing, location and marker gene expression (Sepp et al., 2024). *Foxp2* was expressed evenly across all four embryonic Purkinje cell subtypes, with a small significant enrichment in the early-born, *Foxp1*-high subtype (enrichment in each cluster: *Cdh9*-high subtype FDR=1, *Etv1*-high subtype FDR=1, *Foxp1*-high subtype FDR=0.0185, *Rorb*-high subtype FDR=1) (Fig. S1B). *Foxp1* and *Foxp4* were differentially expressed across mouse Purkinje cell subtypes, with both highly expressed in the early-born, *Foxp1-*high cluster (enrichment in *Foxp1*-high cluster, *Foxp1* FDR=1.2×10^−83^, *Foxp4* FDR=4.07×10^−6^) (Fig. S1B). Together, these findings suggest that FOXP gene co-expression is present in developing human Purkinje cells, with expression levels varying across subtypes and over time.

Having observed similar patterns in FOXP expression in the snRNAseq data between mouse and human, we used immunostaining of mouse cerebellar sections to examine how the transcriptomic variation related to the spatial distribution of FOXP proteins (see Supplementary Materials and Methods). In agreement with the relatively even expression across subtypes in the transcriptomic data, FOXP2 protein expression was observed broadly in Purkinje cells. By embryonic day (E)15.5, FOXP2 was detected in Purkinje cells migrating from the ventricular zone to form the Purkinje cell plate visible at E18.5 and later in the Purkinje cell layer (PCL) (Fig. S1C-G). The only exception we observed was in the most posterior lobule in lateral sections, consistent with previous descriptions of little or no FOXP2 in a small population of Purkinje cells in the most lateral, rostral and ventral region of the cerebellum (Fujita and Sugihara, 2012) (Fig. S1F). Reflecting the differential expression that we had identified across transcriptional defined subtypes, FOXP1 and FOXP4 showed more heterogeneous expression in Purkinje cells at E15.5 (Fig. S1C,D), with FOXP1 only sparsely present in medial sections compared to broader expression in lateral sections. At E18.5, FOXP1^+^ cells were largely found on the outer surface of the cerebellum and no longer colocalised with FOXP2-expressing Purkinje cells within the cerebellum (Fig. S1E). FOXP4 continued to display differential expression between Purkinje cell clusters at E18.5 (Fig. S1E); however, in the postnatal mouse cerebellum, FOXP4 expression was found uniformly across all Purkinje cells (Fig. S1F). Together, the gene expression and immunostaining data confirmed the expression of FOXP2 in developing human and mouse Purkinje cells. The co-expression of other FOXP family genes suggests that FOXP heterodimers may be present, with varying composition possible across subtypes and over time. Our observations also suggest that FOXP4, in addition to previously described FOXP1, shows differential expression across embryonic Purkinje cell subtypes in both mouse and human.

### iPSC-derived cerebellar organoids provide a human model to study FOXP expression in the developing cerebellum

**Fig. 2.**
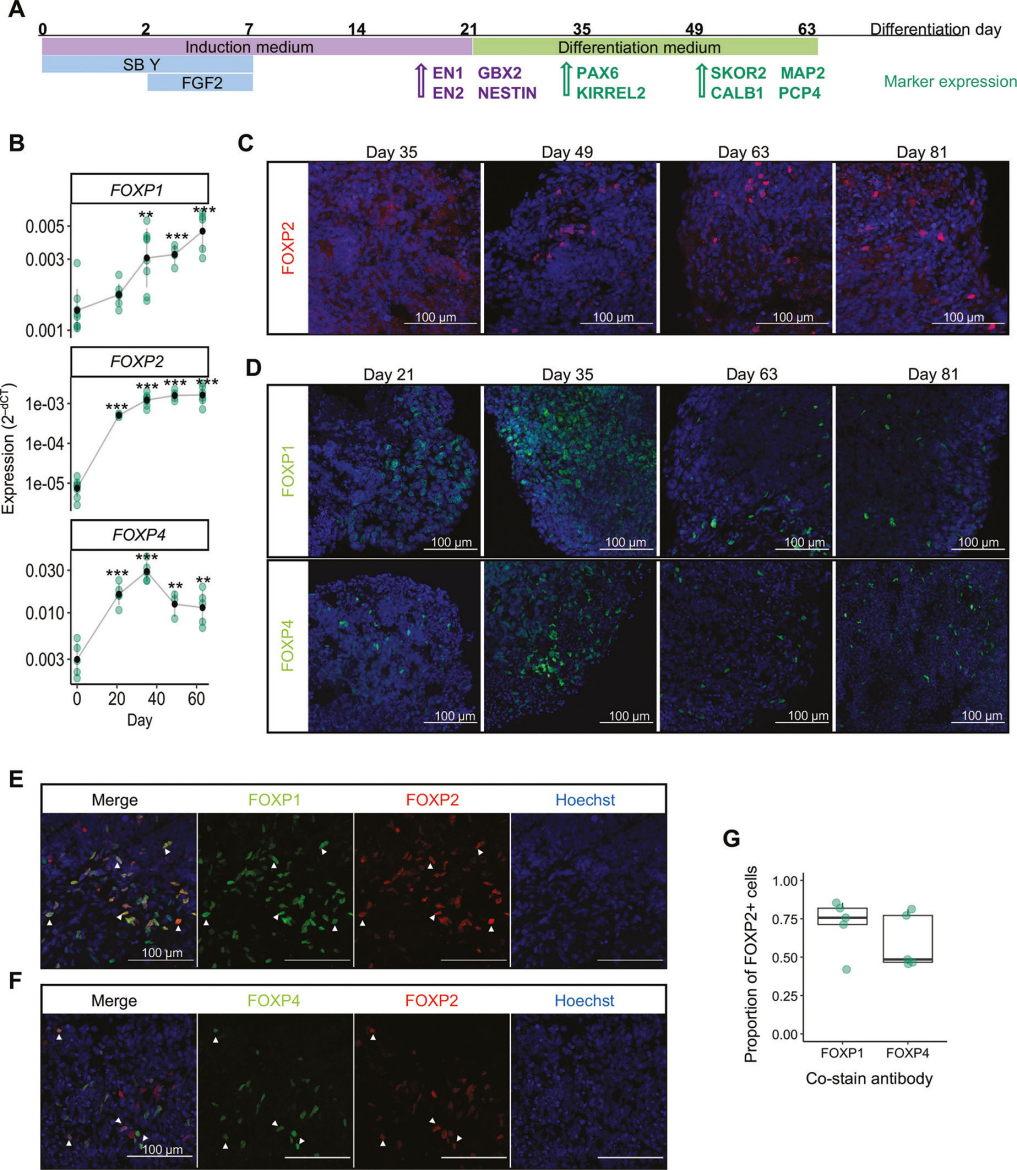
**Cerebellar organoids recapitulate expression of FOXP family genes during differentiation.** (A) Schematic of cerebellar organoid differentiation. Human induced pluripotent stem cell (iPSC) aggregates are patterned towards cerebellar identity using growth factors including fibroblast growth factor 2 (FGF2), TGFβ inhibitor SB431542 (SB), anti-apoptotic factor rock inhibitor Y-27632 (Y) and insulin (component of induction medium). Specific cell lineage markers used to monitor differentiation at corresponding timepoints are indicated. (B) FOXP genes significantly increase in expression during cerebellar organoid development. Expression calculated as 2^−dCT^, relative to housekeeper genes *ACTB* and *GAPDH*. Significant change in expression compared to day 0 determined using an unpaired, two-tailed *t*-test on dCT values. **P<0.01, ***P<0.001. Green points represent individual differentiations (*n*=3-9); black points show mean and error bars representing mean±s.d. (C) Immunostaining against FOXP2 (red) across cerebellar organoid differentiation with consistent detection of FOXP2^+^ cells by day 49. Nuclei were stained with Hoechst (blue). (D) Imaging of FOXP1 and FOXP4 (green) protein expression across cerebellar organoid differentiation. Nuclei were stained with Hoechst (blue). (E,F) Dual staining images showing co-expression of FOXP1 (green; E) and FOXP4 (green; F) with FOXP2 (red), as marked by arrowheads. Nuclei were stained with Hoechst (blue). (G) The colocalisation of FOXP protein staining was quantified as the proportion of FOXP2^+^ cells co-expressing FOXP1 and FOXP4, respectively. Data points represent separate differentiations (*n*=5), calculated from imaging at least three organoid sections per differentiation. Boxes contain the median and span from the first to third percentile. Whiskers indicate the minimum and maximum values, excluding outliers which lie more than 1.5× interquartile range from the box. Scale bars: 100 µm.

Our analysis of transcriptomic data from the developing human cerebellum confirmed enriched expression of FOXP2 in developing human Purkinje cells and suggested that differential expression of FOXP genes contributes to Purkinje cell subtype patterning. Although these patterns could be validated in mouse tissue, we next sought an experimental human model to investigate the function of FOXP2 in the developing human cerebellum. Cerebellar organoids provide an attractive *in vitro* system to study early human cerebellar development, modelling the specification of cerebellar identity and production of cerebellar neurons from both the rhombic lip and ventricular zone lineages (Muguruma et al., 2015; Nayler et al., 2021). Multiple groups have generated cerebellar organoids from different stem cell lines with consistent results, using TGFβ inhibition combined with FGF2 and insulin treatment to direct the differentiation of stem cell aggregates towards cerebellar identity (Chen et al., 2023; Muguruma et al., 2015; Nayler et al., 2021; Silva et al., 2020). To investigate whether cerebellar organoids could be used to investigate FOXP2 in the developing human cerebellum, we first characterised the expression of FOXP family genes throughout differentiation in iPSC-derived cerebellar organoids. Cerebellar organoids were generated from human iPSCs (line AH017-3) according to our established protocol (Tolonen et al., 2024) (Fig. 2A; Fig. S2A,B). By day 35 of the differentiation protocol, organoids contained cells from both cerebellar germinal zones, shown by immunostaining against KIRREL2 labelling the ventricular zone, and PAX6 labelling rhombic lip-derived granule cell progenitors and granule cells, indicating successful differentiation (Tolonen et al., 2024) (Fig. S2A,B). mRNA expression of all three FOXP genes was found to be strongly upregulated within the first 21 days of cerebellar differentiation (Fig. 2B). *FOXP2* and *FOXP4* mRNA expression plateaued from day 49 of differentiation, whereas *FOXP1* mRNA expression continued to increase (Fig. 2B). At a protein level, FOXP2 expression was consistently detected in organoids by day 49 (Fig. 2C). FOXP1 and FOXP4 protein expression was observed from day 21 (Fig. 2D). We also confirmed the differentiation to cerebellar identity and induction of similar FOXP2 expression in cerebellar organoids generated with an alternative iPSC line (WTC11) (Fig. S2C-E). To explore the potential FOXP interactions present in cerebellar organoids, we quantified the co-expression of FOXP proteins at day 63, a timepoint with high FOXP2 expression. FOXP2 was commonly co-expressed with other FOXP proteins: of cells staining positive for FOXP2, 75% were also positive for FOXP1 and 63% were FOXP4 positive (Fig. 2E-G). Together, our analysis of cerebellar organoid samples showed strong induction of *FOXP2* during cerebellar differentiation and indicates that expression of FOXP genes in the early developing cerebellum is recapitulated using iPSC-derived cerebellar organoids. The presence of both double and single FOXP^+^ cells indicates the heterogeneity of FOXP2^+^ cells in cerebellar organoids. This could reflect Purkinje cell subpopulations as described *in vivo* (Sepp et al., 2024; Wizeman et al., 2019) or represent a mixture of different cell types.

### Generation of a *FOXP2* iPSC reporter line facilitates live visualisation and isolation of *FOXP2*^+^ cells in cerebellar organoids

**Fig. 3.**
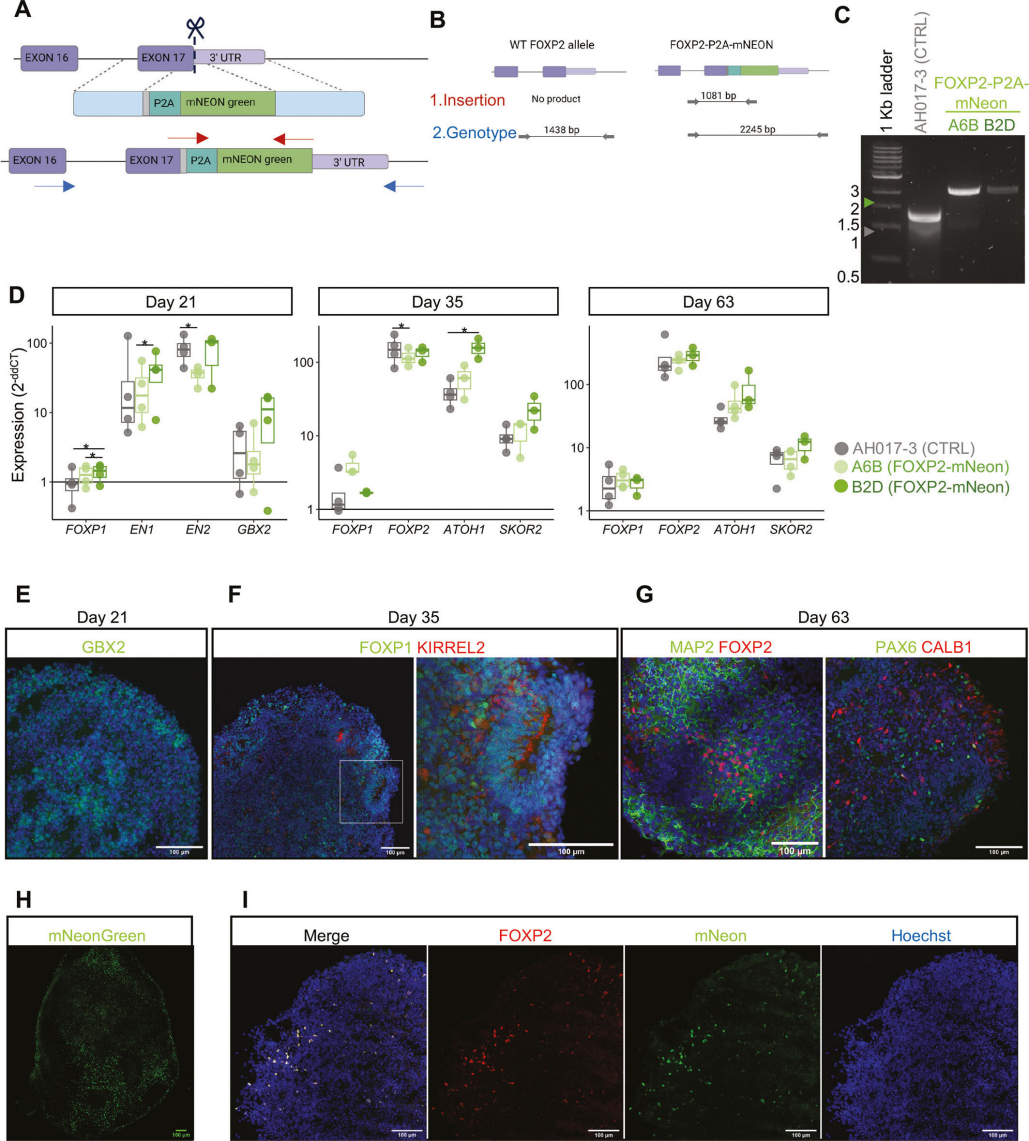
**Generation of FOXP2-mNeon iPSCs and cerebellar organoids.** (A) CRISPR strategy for the generation of a FOXP2-mNeon reporter line. A specific gRNA targets Cas9 (scissors) to the endogenous *FOXP2* stop codon [immediately upstream of the 3′ untranslated region (UTR)]. The donor repair construct contains a P2A-mNeon sequence surrounded by homology arms (light blue) matching the target sequences, indicated by the dashed lines. The result of homology-dependent repair shown below is a FOXP2-P2A-mNeon reporter gene. The position of primers used for screening is indicated by arrows (red, insertion; blue, genotyping). (B) Schematic of PCR products resulting from screening clones, first detecting insertion and then genotyping. (C) Products of the genotyping PCR on a 1% agarose gel from the parental AH017-3 line and two clones with mNeon insertion (A6B and B2D) demonstrate successful gene editing. CTRL, control. (D) Expression of cerebellar markers at days 21, 35 and 63 of cerebellar organoid differentiation using two FOXP2-mNeon clones (A6B, light green; B2D, dark green) and the parental line (AH017-3, grey) by reverse transcription quantitative PCR. Expression calculated relative to that in iPSCs (day 0) as 2^−ddCT^. Dots indicate individual differentiations (*n*=3-4). Boxes show contain the median and span from the first to third percentile. Whiskers indicate the minimum and maximum values, excluding outliers which lie more than 1.5× interquartile range from the box. Significant differences in gene expression between lines at each timepoint were tested for using a one-way ANOVA followed by post-hoc Tukey's HSD. *P<0.05. (E) Expression of hindbrain marker GBX2 (green) at day 21 of cerebellar differentiation using FOXP2-mNeon clone B2D. Nuclei were stained with Hoechst (blue). (F) Expression of markers of the cerebellar ventricular zone (FOXP1, green; KIRREL2, red) at day 35 of cerebellar differentiation using FOXP2-mNeon clone B2D. Nuclei were stained with Hoechst (blue). (G) Expression of markers of Purkinje cells (FOXP2, red; CALB1, red), granule neurons (PAX6, green) and mature neurons (MAP2, green) at day 63 of cerebellar differentiation using FOXP2-mNeon clone B2D. Nuclei were stained with Hoechst (blue). (H) Live-cell imaging of mNeon fluorescence in a day 58 cerebellar organoid generated from FOXP2-mNeon clone B2D, showing distribution of FOXP2^+^ cells. (I) Immunofluorescent staining of FOXP2 (red) and mNeon (green) confirms accurate co-expression in sections of a day 63 cerebellar organoid generated using FOXP2-mNeon clone B2D. Nuclei were stained with Hoechst (blue). Scale bars: 100 µm.

Cerebellar organoids do not recapitulate the spatial organisation of the developing cerebellum and therefore showed a heterogeneous distribution of FOXP2-expressing cells across the organoid (Fig. 2C) compared to the distinctive staining pattern observed in tissue sections of the developing mouse cerebellum (Fig. S1C-G). This lack of spatial organisation makes it hard to identify specific cerebellar cell types purely by their position in the organoid and typically requires analysis of marker gene expression in fixed samples. Previous work in our laboratory characterised day 90 cerebellar organoids using scRNAseq, identifying major cerebellar cell types (Nayler et al., 2021). However, relatively low numbers of cells were captured, including few Purkinje cells and *FOXP2*^+^ cells (1653 total cells, 107 Purkinje cells, 247 *FOXP2*^+^ cells) (Fig. S2F,G). Therefore, we chose a different approach to define the cell types expressing FOXP2 across time in cerebellar organoid differentiation. To facilitate the study of *FOXP2*-expressing cells, including live visualising this population, we generated an iPSC line containing a fluorescent reporter for *FOXP2* expression. We targeted the endogenous *FOXP2* locus to achieve an accurate readout of *FOXP2* expression. mNeonGreen (hereafter, mNeon) was selected for bright fluorescent properties given the relatively weak expression of an endogenous promoter (Shaner et al., 2013). CRISPR-mediated gene editing was employed using a single-stranded DNA donor, encoding a self-cleaving peptide P2A (Donnelly et al., 2001; Wang et al., 2015) and the fluorescent protein mNeon flanked by homology arms (Fig. 3A). iPSC clones with successful homozygous integration of the mNeon sequence were screened for by PCR (Fig. 3B,C; Fig. S3A-C), and accurate insertion of the reporter gene was confirmed by Sanger sequencing (Fig. S3D). We selected two clones (A6B, B2D) with homozygous insertion of mNeon into the *FOXP2* locus for future experiments.

No mutations resulting from the CRISPR process were detected upon sequencing of the top six high-risk off-target sites predicted by online tools CCTop and CRISPOR for the gRNA used (Fig. S4). In addition, FOXP2-mNeon clones showed equivalent gene dosage to control iPSC AH017-3 by single-nucleotide polymorphism array (SNP) array [noting a loss of heterozygosity without copy number loss on Chr17p in the parental AH017-3 working stock (Fig. S5)]. FOXP2-mNeon clones displayed typical iPSC morphology and showed widespread expression of pluripotency markers NANOG and TRA-1-60 when assessed by flow cytometry (Fig. S6A,B). Moreover, the generated reporter line iPSCs could be differentiated successfully into all three germ lineages following respective directed differentiation, confirming their pluripotency (Fig. S6C). Together, these results indicate that the generated iPSC reporter line maintained normal stem cell characteristics following the CRISPR editing and clone selection process.

Next, we tested the capability of the newly generated *FOXP2* reporter line to differentiate reliably into cerebellar organoids. Both FOXP2-mNeon clones successfully generated cerebellar organoids with typical growth, morphology and induction of cerebellar genes used for quality control (Fig. 3D). Markers of the midbrain-hindbrain boundary were present by day 21, with significant upregulation of *EN1* and *EN2* (unpaired, one-tailed *t*-test on dCT values, day 21 vs iPSC; A6B *P*-values: *EN1=*1.30×10^−4^, *EN2=*6.16×10^−6^, *GBX2=*0.114, *FOXP1=*0.102; B2D *P*-values: *EN1=*6.43×10^−4^, *EN2=3.23*×10^−4^, *GBX2=*0.0303, *FOXP1=*0.0825) (Fig. S7A) and positive staining for GBX2 (Fig. 3E). At day 35 and 63, reporter line-derived cerebellar organoids expressed markers for progenitors and neurons of both key cerebellar neuron lineages: granule cells (*ATOH1*, *PAX6*) and Purkinje cells (*KIRREL2*, *SKOR2*, *CALB1*) (Fig. 3F,G; Fig. S7B). For both FOXP2-mNeon clones, there was significant induction of Purkinje cell (*FOXP1*, *FOXP2*, *SKOR2*) and granule cell (*ATOH1*) marker genes, at day 35 and day 63 compared to those in iPSCs as measured by quantitative PCR (qPCR) (unpaired, one-tailed *t*-test on dCT values, day 35 vs iPSC or day 63 vs iPSC, *P*<0.01 for all genes) (Fig. S7B). Organoids generated from both FOXP2-mNeon reporter line clones showed very similar expression of profiled markers compared to that in the parental line (Fig. 3D). At day 63, organoids widely expressed MAP2, indicating a high proportion of differentiated neurons (Fig. 3G). Following generation of FOXP2-mNeon cerebellar organoids, mNeon expression could be successfully visualised by live-cell fluorescent microscopy (Fig. 3H). Immunofluorescent staining of fixed organoid sections showed tight co-expression of the mNeon reporter with FOXP2 in both reporter line clones (Fig. 3I; Fig. S7C), verifying the accuracy of mNeon signal as a readout of endogenous FOXP2 expression. The subcellular localisation of mNeon was observed as nuclear in both live-cell microscopy and immunostaining (Fig. 3H,I; Fig. S7C). This suggested that the fluorescent mNeon tag was not separated from the nuclear-localised FOXP2 by the intended P2A-mediated cleavage. The presence of a FOXP2-mNeon fusion protein, instead of separated FOXP2 and mNeon proteins, was confirmed by western blotting (Fig. S8). Together, these results demonstrate that the generated FOXP2-mNeon iPSC lines could be differentiated reliably to cerebellar identity, including a *FOXP2*^+^ population accurately labelled live by mNeon fluorescence.

### Transcriptomic analysis of *FOXP2*-expressing cells in cerebellar organoids reveals Purkinje cell and CN neuron identities

To further characterise the cellular identity of *FOXP2*^+^ cells in the cerebellar organoids, we performed RNA sequencing on sorted populations of mNeon^+^ and mNeon^−^ cells from differentiated *FOXP2* reporter line organoids (FOXP2-mNeon clone B2D). FOXP2-mNeon cerebellar organoids were dissociated to single cells at day 63 of differentiation, around the peak of FOXP2^+^ cells detected by immunostaining (Fig. 2C). Flow cytometry was used to sort live cells into mNeon^+^ and mNeon^−^ populations, with ∼3% of live cells in the mNeon^+^ gate (Fig. S9). RNA was extracted from the sorted cells, and bulk RNA sequencing was performed on mNeon^+^ and mNeon^−^ populations from four differentiation batches.

**Fig. 4.**
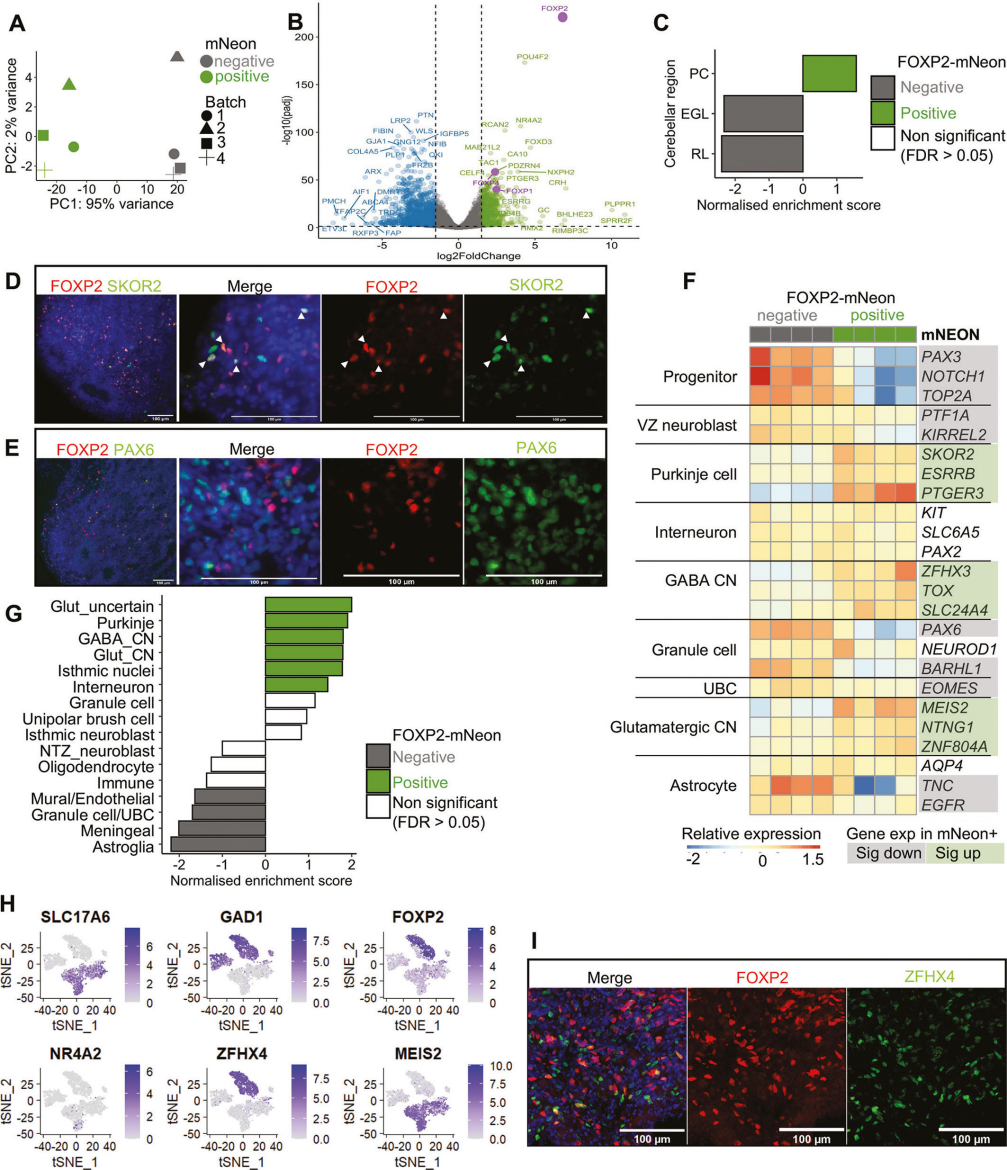
**Transcriptomic characterisation of mNeon-sorted populations identifies *FOXP2* expression in Purkinje cells and cerebellar nuclei neurons in cerebellar organoids.** (A) mNeon-positive (green) and mNeon-negative (grey) populations showed clear separation across the *x*-axis, with principal component (PC)1 accounting for the majority of variance between samples. Individual differentiations shown by different shapes contribute a small amount of variance across PC2 (*y*-axis). (B) Differentially expressed genes in the mNeon-positive populations are displayed on volcano plot. Genes increased in mNeon-positive samples are shown in green [log2FC)>1.5, adjusted *P*-value (*P*adj)<0.05], including *FOXP2* (log2FC=6.82, *P*adj=1.75×10^−224^), *FOXP1* (log2FC=2.4, *P*adj=5.2×10^−41^) and *FOXP4* (log2FC=2.3, *P*adj=1.61×10^−58^). Genes significantly decreased in mNeon^+^ samples are shown in blue (log2FC<−1.5, *P*adj<0.05). Differential gene expression Wald test with Benjamini–Hochberg multiple testing adjustment using DESeq2. (C) Enrichment of genes differentially expressed between fetal human cerebellar regions (Aldinger et al., 2021) (Table S4) in FOXP2-mNeon^+^ and FOXP2-mNeon^−^ cerebellar organoid populations. Gene set enrichment analysis (GSEA) calculated a normalised enrichment score and FDR for each gene set. EGL, external granule layer; PC, Purkinje cell layer; RL, rhombic lip. (D) Immunostaining for FOXP2 (red) with Purkinje cell marker SKOR2 (green) using day 63 cerebellar organoid sections. Arrowheads indicate co-expression. Nuclei were stained with Hoechst (blue). (E) Immunostaining for FOXP2 (red) with granule cell marker PAX6 (green) of cerebellar organoid sections (day 49) did not identify co-expression. Nuclei were stained with Hoechst (blue). (F) Expression of specific cell type markers across organoid populations indicated that the *FOXP2*^+^ cerebellar organoid cells contain a mixture of Purkinje cell and cerebellar nuclei (CN) neurons. Colour scale shows expression scaled for each gene. Genes highlighted have *P*adj<0.05 and are increased (green, log2FC>0) or decreased (grey, log2FC<0) in the *FOXP2*^+^ population. UBC, unipolar brush cells; VZ, ventricular zone. (G) The distribution of genes specifically expressed in distinct cerebellar cell types was assessed across cerebellar organoids using GSEA (Table S5). Gene sets significantly enriched (FDR<0.05) in the *FOXP2*^+^ or *FOXP2^−^* cerebellar organoid population are coloured in green and grey, respectively. CN, cerebellar nuclei; Glut, glutamatergic; NTZ, nuclear transitory zone; UBC, unipolar brush cell. (H) Plotting published snRNAseq data from human cerebellar nuclei neurons shows their division into glutamatergic excitatory (*SLC17A6*) and GABAergic inhibitory (*GAD1*) clusters (Kebschull et al., 2020). *FOXP2* expression is enriched in an *GAD1^+^* inhibitory cluster, but also shows broader expression. Expression of selected genes upregulated in the *FOXP2*^+^ cerebellar organoid population (*NR4A2*, *ZFHX4*, *MEIS2*) are shown across the same dataset. (I) Immunostaining of day 63 cerebellar organoid, using antibodies against *FOXP2* (red) and *ZFHX4* (green). Nuclei were stained with Hoechst (blue). Scale bars: 100 µm.

Principal component (PC) analysis revealed clear separation of the mNeon^+^ and mNeon^−^ samples around PC1, with this parameter responsible for 95% variance compared to a very small effect between individual differentiations (Fig. 4A). Differential expression analysis confirmed the isolation of the *FOXP2*-expressing cells in the mNeon^+^ population (Fig. 4B). In total, 7644 genes were identified as differentially expressed below the adjusted *P*-value (*P*adj) threshold of 0.05, with 3655 genes increased and 3989 decreased in the mNeon^+^ population relative to those in the mNeon^−^ cells. In agreement with our immunostaining results (Fig. 2E,F), *FOXP1* and *FOXP4* expression was significantly enriched in the mNeon^+^, *FOXP2*-expressing population (Fig. 4B).

To assess the cell type identity most closely represented by the *FOXP2*^+^ population, we compared the distribution of marker genes across the flow cytometry-sorted cerebellar organoid samples. Gene set enrichment analysis (GSEA) was performed using differentially expressed genes in fetal human cerebellar layers (Aldinger et al., 2021) (Table S4). The *FOXP2*^+^ population showed significant enrichment for genes distinguishing the PCL, while the *FOXP2*^−^ population showed significant enrichment for genes highest in the rhombic lip and external granule cell layer (Fig. 4C). These results are consistent with *FOXP2* marking Purkinje cells and suggest that the remaining organoid population contained proliferative progenitors, including granule cell progenitors, and non-neuronal cells. Immunostaining confirmed overlap in protein expression of the Purkinje cell-specific marker SKOR2 within the FOXP2^+^ population and lack of colocalisation of FOXP2 with PAX6, a marker of granule cell precursors and neurons (Fig. 4D,E; Fig. S10). In other bioinformatic analysis, we confirmed high expression of *FOXP2* in Purkinje cells that were differentiated through a recent alternative protocol for generation of cerebellar organoids from human iPSCs (Atamian et al., 2024) (Fig. S11).

To refine the identity of the *FOXP2*^+^ cerebellar organoid population further, we examined the expression of selected markers of different cerebellar cell types (Fig. 4F). We selected marker genes from a cross-species snRNAseq characterisation of cerebellar development, in particular verifying their cell type specificity in the developing human cerebellum (Sepp et al., 2024) (Fig. S12). The *FOXP2*^+^ population showed significantly increased expression of markers of Purkinje cells, particularly those present in earlier stages of cerebellar development (e.g. *SKOR2*, *ESRRB*, *PTGER3*, *LHX1*, *LHX5*, *TFAP2A*). Genes associated with progenitors (*PAX3*, *NOTCH1*, *TOP2A*) and ventricular zone neuroblast (*KIRREL2*, *PTF1A*) were significantly decreased in the *FOXP2*^+^ population, indicating maturity of the *FOXP2*^+^ population as differentiated neurons in contrast to progenitors. Other cerebellar cell lineages were excluded from the *FOXP2*^+^ population, based on decreased expression of markers for granule cells (*PAX6*, *ATOH1*, *BARHL1*) and unipolar brush cells (*EOMES*, *LMX1A*), and no significant difference in markers for GABAergic interneurons (*PAX2*, *KIT*, *SLC6A5*). However, several genes associated with both glutamatergic (*SLC17A6*, *MEIS2*, *NTNG1*, *ZNF804A*) and GABAergic (*ZFHX3*, *ZFHX4*, *TOX*, *SLC24A4*) neurons found in the CN were also found to be enriched in the *FOXP2*^+^ organoid population. To expand from our short curated list of cell type markers, we calculated differentially expressed genes for each cluster present in the Sepp et al. (2024) dataset to determine a list of genes specific to each cerebellar cell type, and these marker gene lists were used in GSEA (Table S5). In support of our initial conclusions, the *FOXP2*^+^ population showed significant enrichment for markers of Purkinje cells (Fig. 4G). GSEA also identified upregulation of markers of GABAergic and glutamatergic CN, and to a lesser extent interneurons, in the *FOXP2*^+^ population (Fig. 4G). In addition, there was significant enrichment for markers of isthmic nuclei neurons (genes including *NR4A2* and *SCG2*) and a cluster labelled as ‘glutamatergic_uncertain’ (genes including *VSNL1*, *RIMS1*, *FSTL5*), both clusters from the nuclear transitory zone lineage, which gives rise to glutamatergic CN and isthmic nuclei neurons (Sepp et al., 2024).

Our results suggest that CN neurons expressing *FOXP2* were among the *FOXP2*^+^ population in cerebellar organoids. Supporting the expression of *FOXP2* in CN, we also detected sparse FOXP2-expressing cells in the cerebellar white matter in mouse immunostained cerebellar sections (Fig. S1G), in line with previous descriptions (Ferland et al., 2003; Lai et al., 2003). However, in our earlier analysis of snRNAseq data from the developing human cerebellum, we did not detect substantial FOXP2 expression in captured CN neurons (Fig. 1B). Therefore, we made use of a published snRNAseq dataset of isolated human CN neurons (Kebschull et al., 2020) to evaluate whether *FOXP2* is also expressed in the human CN and, if so, in which cell types. The CN contain excitatory (primarily glutamatergic) and inhibitory (primarily GABAergic) neurons that can be distinguished by expression of *SLC17A6* and *GAD1*, respectively (Kebschull et al., 2020) (Fig. 4H). We found that expression of *FOXP2* was highest in a cluster of inhibitory CN neurons, annotated as ‘inferior olive projection neurons’, but was also widely expressed at a lower level across excitatory CN clusters (Fig. 4H). Next, we plotted the expression of genes enriched in the *FOXP2*^+^ organoid population associated with the isthmic nuclei (*NR4A2*), GABAergic CN neurons (*ZFHX4*) and glutamatergic CN neurons (*MEIS2*) in the human CN dataset. *ZFHX4* showed a similar profile to *FOXP2*, with enrichment in the same inhibitory neuron cluster, whereas *MEIS2* and *NR4A2* were higher in excitatory neurons (Fig. 4H). Immunostaining was performed to confirm the co-expression of one of these CN markers, ZFHX4, with FOXP2 in cerebellar organoids (Fig. 4I; Fig. S10). Together, our results suggest that multiple cerebellar cell types express FOXP2 during early human development, most notably Purkinje cells and CN, and these cell types were recapitulated in iPSC-derived cerebellar organoids.

### *FOXP2*^+^ cells show enrichment for pathways relating to neuronal activity

**Fig. 5.**
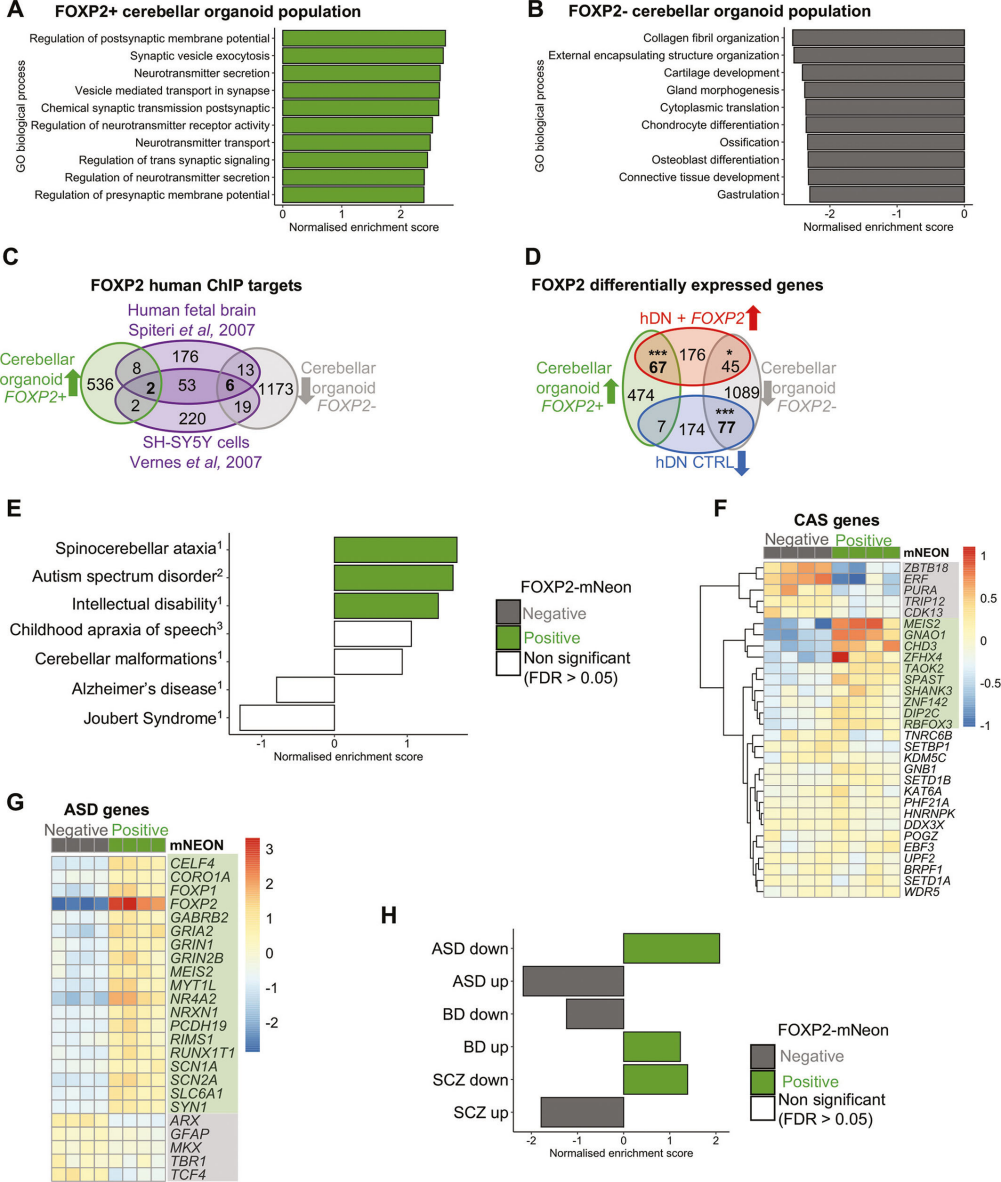
**The *FOXP2*^+^ population in cerebellar organoids is enriched for expression of pathways relating to synaptic activity and for genes associated with certain neurodevelopmental disorders.** (A,B) Upregulated (A) and downregulated (B) biological processes in the *FOXP2*^+^ cerebellar organoid population as calculated by GSEA. Normalised enrichment scores for the top ten Gene Ontology (GO) biological process terms are shown. (C) Differentially expressed genes between *FOXP2*^+^ (green) and *FOXP2*^−^ (grey) cerebellar organoid populations were compared to FOXP2 targets identified in published FOXP2 chromatin immunoprecipitation studies on neuronal samples (Vernes et al., 2007; Spiteri et al., 2007), and overlaps are shown (purple). Overlapping genes are listed in Table S6. (D) Differentially expressed genes were compared between *FOXP2*^+^ (green, left) and *FOXP2*^−^ (grey, right) cerebellar organoid populations and human differentiated neurons with *FOXP2* (red, top) and without *FOXP2* (blue, bottom) expression (Hickey et al., 2019). Significance of gene set overlap was determined using a Fisher's exact test. *P<10^−5^, ***P<10^−26^. Overlapping genes are listed in Table S7. (E) GSEA identified enrichment of genes associated with spinocerebellar ataxia, autism spectrum disorder (ASD) and intellectual disability in the *FOXP2*^+^ cerebellar organoid population. Gene sets were collated from multiple sources and are listed in Table S8: ^1^previous testing in human cerebellar scRNAseq (Aldinger et al., 2021); ^2^SFARI class 1 risk genes; ^3^published childhood apraxia of speech (CAS) genes (Kaspi et al., 2022; Eising et al., 2019; Hildebrand et al., 2020). Bars are coloured according to significance (FDR<0.05) and direction of enrichment. (F) Expression of CAS-associated genes across *FOXP2*^+^ and *FOXP2*^−^ cerebellar organoid populations. Genes significantly differentially expressed (*P*adj<0.05) between FOXP2-sorted populations are highlighted in green (increased) or grey (decreased). (G) Expression of ASD-associated genes that showed significant differential expression (*P*adj<0.05) between *FOXP2*^+^ and *FOXP2*^−^ cerebellar organoid populations. Genes highlighted according to change in the *FOXP2*^+^ population in green (increased) or grey (decreased). (H) Enrichment of genes differentially expressed in post-mortem cortex samples from individuals with ASD, bipolar disorder (BD) and schizophrenia (SCZ). Differentially expressed genes identified by Gandal et al. (2018) (Table S9); genes that were significantly increased in affected individuals compared to unaffected controls were classified as ‘up’, and those that were decreased in affected individuals compared to unaffected controls were classified as ‘down’. GSEA was performed to calculate a normalised enrichment score and FDR for each gene set across *FOXP2*^+^ and *FOXP2*^−^ organoid populations.

FOXP2 acts as a transcriptional regulator, capable of transcriptional repression or activation, depending on interacting partners (Hickey et al., 2019; Li et al., 2004; Shu et al., 2001), and therefore influences a range of cellular processes. To investigate potential downstream pathways of FOXP2 in the developing human cerebellum, we performed GSEA using biological process Gene Ontology (GO) terms. GSEA identified enrichment for terms related to synaptic processes, neuronal activity and signal transduction in the *FOXP2*^+^ population (Fig. 5A). These pathways included upregulation of genes such as *GLRA*2, *CELF4*, *GRIN3A* and *GABRA3* for regulation of membrane potential, and synaptic vesicle components including synaptotagmins (*SYT7*, *SYT12*, *SYT5*), synaptophysin (*SYP*), synapsin (*SYN1*) and synaptic vesicle glycoproteins (*SV2C*, *SV2A*). Terms identified for the *FOXP2*^−^ population were more varied and related to extracellular matrix, cytoplasmic translation and connective tissue development (Fig. 5B). As the *FOXP2*^+^ and *FOXP2*^−^ populations differ in cell type identity (Fig. 4), the observed differences in enriched cellular processes might be influenced by the presence of different cell types in addition to differences in *FOXP2* expression. Furthermore, our approach can only identify pathways upregulated in *FOXP2*^+^ cells and cannot distinguish which are directly regulated by FOXP2. However, previous studies in non-cerebellar cells have identified similar pathways related to neurogenesis and synaptic signalling regulated by FOXP2 (Oswald et al., 2017; Spiteri et al., 2007; Vernes et al., 2007, 2011).

In order to focus on transcriptional changes, which could be directly mediated by FOXP2, as opposed to differences in cell type identity, genes differentially expressed across organoid populations were compared to known FOXP2 targets. Several published studies have identified FOXP2 targets using chromatin immunoprecipitation (ChIP). However, there is very limited overlap in the results of these studies and, thus, currently no consensus list of FOXP2-regulated genes exists (den Hoed et al., 2021). As no FOXP2-ChIP studies have been performed on isolated cerebellar tissue, we focused on comparison with human neuronal studies, using human fetal brain (Spiteri et al., 2007) and a human neuroblastoma cell line SH-SY5Y stably expressing *FOXP2* (Vernes et al., 2007). When selecting cerebellar organoid differentially expressed genes for this analysis, we applied a stricter threshold of an absolute log2FC in expression greater than 1.5 and *P*adj below 0.05 to focus on the genes with most specific expression to one population. This yielded a list containing 578 genes increased and 1253 genes decreased in the *FOXP2*^+^ population. From the two FOXP2-ChIP studies, only 61 common targets were identified; two of these were significantly increased (*FOXD3*, *PDZRN*) in the *FOXP2*^+^ population in cerebellar organoids, and six were found to be significantly decreased (*PTN*, *LRP2*, *COL4A5*, *HTRA1*, *LRP4*, *NOTCH2*) (Fig. 5C; Table S6). We also examined the expression of a broader list of genes shown to be downstream of FOXP2 regulation in human neurons. These genes were identified by differential gene expression analysis of neurons differentiated *in vitro* from human neural progenitors that do not express *FOXP2* (control) or with exogenous *FOXP2* expression (Hickey et al., 2019). Interestingly, a significant number of the genes present in both datasets showed the same pattern in expression when comparing between *FOXP2*^+^ and *FOXP2*^−^ cells: 67 genes were significantly upregulated in the *FOXP2*^+^ vs *FOXP2*^−^ cells of cerebellar organoids and in *FOXP2*^+^ human differentiated neurons vs control human differentiated neurons, and 77 genes were decreased in both (Fig. 5D; Table S7). These results suggest that a set of genes might be commonly regulated by FOXP2 in neuronal differentiation across brain regions.

### Genes associated with NDDs are enriched in *FOXP2*^+^ cells

Having explored pathways active in normal development, we next sought to identify whether the *FOXP2*^+^ cerebellar population was likely to be affected in disease by examining the expression of disease-associated genes across *FOXP2*^+^ and *FOXP2*^−^ cerebellar organoid populations. Aberrant cerebellar development is linked to a range of NDDs including ASD (Becker and Stoodley, 2013; Sathyanesan et al., 2019; Sydnor and Aldinger, 2021). Moreover, developmental abnormalities also likely contribute to cerebellar diseases that are classically regarded as neurodegenerative conditions such as spinocerebellar ataxia (SCA) (Edamakanti et al., 2018; Leto et al., 2016; Serra et al., 2006). In addition, we evaluated risk genes for childhood apraxia of speech (CAS), the core phenotype of the *FOXP2*-related speech and language disorder (Morgan et al., 2016). Employing GSEA using gene sets for cerebellar disorders, as previously examined in human fetal cerebellum (Aldinger et al., 2021), and a list of CAS-risk genes (Eising et al., 2019; Hildebrand et al., 2020; Kaspi et al., 2022), we found significant enrichment for genes associated with specific disorders including SCA, ASD and intellectual disability in the *FOXP2*^+^ cerebellar organoid population (Fig. 5E; Table S8). There was also enrichment, albeit non-significant, for CAS-associated genes as well as cerebellar malformations (Fig. 5E). As expected, no enrichment was identified in genes associated with Alzheimer's disease, a neurodegenerative disease not linked to cerebellar development, and Joubert syndrome, a neurodevelopmental ciliopathy. Joubert syndrome genes have previously been shown to be enriched in the ciliated cell population of the cerebellar organoids (Nayler et al., 2021).

In addition to FOXP2, further monogenic causes of CAS have been identified in a series of sequencing studies of affected probands and their families, offering more insight into the gene networks regulating speech (Eising et al., 2019; Hildebrand et al., 2020; Kaspi et al., 2022). We further examined the expression of individual CAS genes in *FOXP2*^+^ cerebellar cells to identify co-expressed genes that might act in cooperation with *FOXP2* in the early cerebellum. Five genes were significantly decreased, and ten genes were significantly increased, in the *FOXP2*^+^ population (*P*adj<0.05) (Fig. 5F). Interestingly, CAS genes with increased expression included several genes expressed in the CN such as *MEIS2*, *ZFHX4* and *RBFOX3* (Fig. 5F).

Although *FOXP1* has been repeatedly associated with ASD, including the presence of ASD symptoms within the definition of FOXP1 syndrome (Lozano et al., 2021), the association of *FOXP2* and ASD has been less certain, with both positive and negative reports (Co et al., 2020). Interestingly, we found that ASD-associated genes that were differentially expressed between the *FOXP2*^+^ and *FOXP2*^−^ organoid populations included *FOXP1* as well as several genes involved in neuronal activity, particularly synaptic transmission (*GABRB1*, *GRIA2*, *GRIN1*, *GRIN2B*, *SCN1A*, *SCN2A*, *SLC6A1*) (Fig. 5G). Given the strong enrichment for ASD-associated genes in the *FOXP2*^+^ cerebellar organoid population, we also examined cerebellar organoid expression of genes with altered expression in ASD, using an additional dataset profiling differentially expressed genes in post-mortem brains of individuals with neurological disorders (Gandal et al., 2018) (Fig. 5H; Table S9). We found that genes that were decreased in individuals with ASD were significantly enriched in the *FOXP2*^+^ organoid population, whereas genes that were upregulated in post-mortem brains of individuals with ASD were enriched in the *FOXP2*^−^ population in the cerebellar organoids (Fig. 5H). Interestingly, genes increased in individuals with bipolar disorder and genes decreased in individuals with schizophrenia were also significantly enriched in the *FOXP2*^+^ organoid population, while genes decreased in individuals with bipolar disorder and genes increased in individuals with schizophrenia were enriched in the *FOXP2*^−^ organoid population (Fig. 5H).

Together, our findings suggest that *FOXP2*^+^ cell populations are particularly vulnerable to distinct NDDs and highlight specific gene networks associated with this vulnerability. Moreover, our results underscore the utility of the cerebellar organoid model to further study disease mechanisms in vulnerable cerebellar cell types in these disorders.

## DISCUSSION

In this work, we used cerebellar organoids as a model to study the expression of *FOXP2* in the developing human cerebellum. We successfully generated a fluorescent iPSC reporter line to visualise *FOXP2* expression live. Differentiation of this FOXP2-mNeon iPSC line into cerebellar organoids facilitated detailed characterisation of the *FOXP2*^+^ population from among the many organoid cell types. We identified features of Purkinje cells and CN neurons in the *FOXP2*^+^ cerebellar organoid population by comparison to published snRNAseq human cerebellar datasets. The *FOXP2*^+^ cerebellar population showed enrichment for molecular pathways related to neurogenesis and synaptic signalling. Moreover, we identified enrichment of a significant number of disease-associated genes in the *FOXP2*^+^ cerebellar organoid cells. Together, employing a cerebellar organoid model allowed us to profile *FOXP2*^+^ cells in the early developing human cerebellum and highlights these cell types as important for understanding multiple NDDs.

Our findings demonstrate the potential of cerebellar organoids to model early human cerebellar development and disease. We show that cerebellar organoids provide a reliable, human-specific system to examine the expression of FOXP proteins across early cerebellar development. The expression of *FOXP1*, *FOXP2* and *FOXP4* in the human cerebellum and in cerebellar organoids showed similarities to previous descriptions of Foxp gene expression in mouse development (Ferland et al., 2003; Lai et al., 2003; Tam et al., 2011). Our analysis found *FOXP2* to be widely expressed across embryonic mouse and human Purkinje cells, with a slight enrichment in early-born subtypes. We saw greater variation in the distribution of *FOXP1*, with *FOXP1* expression significantly higher in the human early-born Purkinje cell subtype, mirroring multiple studies in mouse and human identifying high *Foxp1* in early-born lateral Purkinje cell clusters (Sepp et al., 2024; Wizeman et al., 2019). In comparison to other FOXP genes, *FOXP4* was expressed in a much lower proportion of fetal human Purkinje cells but, again, showed higher expression in the human early-born Purkinje cell subtype. We also observed a distinct spatial distribution of FOXP4 across Purkinje cells in the developing mouse cerebellum and found enrichment in the early-born, *Foxp1*-high subtype of mouse embryonic Purkinje cells. In agreement with a recent report (Khouri-Farah et al., 2025), our findings suggest that, similar to FOXP1, FOXP4 differs in expression across embryonic Purkinje cell subtypes. In cerebellar organoids, we found that FOXP2 was frequently co-expressed with other FOXP proteins, but we also identified the presence of single FOXP^+^ cells. We therefore hypothesise that cerebellar organoids contain a heterogeneous FOXP2^+^ population, which, among other cerebellar cell types, may contain multiple Purkinje cell subtypes reflective of *in vivo* developmental processes. In the future, the FOXP2-mNeon reporter line could enable live-cell imaging and electrophysiology of developing FOXP2^+^ Purkinje cells and CN in cerebellar organoids. Our findings draw attention to the expression of *FOXP2* in human CN neurons, as captured in snRNAseq of human CN (Kebschull et al., 2020) and the recapitulation of this cell type-specific expression in cerebellar organoids (in this study and Nayler et al., 2021). Histological characterisation of FOXP2 expression in human CN across development is required to resolve discrepancies in expression or absence in human CN neurons described by different snRNAseq studies. In mouse, histological studies have previously reported FOXP2 expression in the CN; however, the focus of FOXP2 functional studies has largely been on Purkinje cells (Ferland et al., 2003; Fujita and Sugihara, 2012; Lai et al., 2003). Along with *FOXP2*, we found that additional genes identified as monogenic causes of CAS such as *ZFHX4* are expressed in the same human CN populations. The CN act to transmit information out from the cerebellum to other brain regions and also have an important feedback role in the inferior olive-cerebellar inhibitory circuits (Kebschull et al., 2023; Leto et al., 2016). Our findings warrant further research into the role of these neurons in studies of FOXP2 disruption and NDDs more generally.

Beyond speech and language disorders, we found enrichment of genes associated with other NDDs in the *FOXP2*^+^ cerebellar organoid population. Our findings in the human cerebellar organoids are consistent with an enrichment of SCA and ASD genes observed in Purkinje cell clusters of human developing cerebellum (Aldinger et al., 2021), underscoring the power of the cerebellar organoids in modelling relevant developing human cell populations that are vulnerable in disease. In particular, ASD-risk genes and genes downregulated in post-mortem brains of individuals with ASD were enriched in the *FOXP2*^+^ population, whereas genes upregulated in ASD were lower in the *FOXP2*^+^ population. This result may reflect a decreased population of specific cell types, such as Purkinje cells or other GABAergic neurons sharing similar expression profiles, or an altered phenotype such as reduced neuronal activity in ASD. Excitatory-inhibitory imbalance is a proposed mechanism underlying ASD and has been investigated in the context of the cerebral cortex and its organoid models, but remains incompletely understood in the cerebellum (Bruining et al., 2020; Sydnor and Aldinger, 2021). We propose that cerebellar organoids offer a promising model to understand the mechanisms of different genetic contributors to ASD during relevant developmental windows. Notably, the strong expression of the ASD-associated *FOXP1* gene in specific human Purkinje cell subtypes and CN neurons calls for further investigation into the function of FOXP1 in these cell types during normal development as well as in NDDs. To determine the role of FOXP genes in NDDs in the cerebellum, CRISPR gene editing could be used to disrupt expression in addition to labelling expression as used in this study.

This study has a number of limitations. First, although the generation of a reporter line provided a useful tool, the use of a single parental line and, subsequently, single clone of the FOXP2-mNeon reporter line limits the biological variation captured in our experiments, and results may be influenced by unknown bias of this genetic background. To mitigate this concern, we verified the generation of cerebellar organoids from a male iPSC line (WTC11) and observed FOXP2 expression consistent with our results using the female line AH017-3. In the future, use of multiple iPSC lines and reporter line clones would improve the robustness of our results as recommended by Designing and Reporting In Vitro Experiments Responsibly (DRIVER) guidelines. Another limitation of our study was the correlative analysis of potential FOXP2 target genes. In the future, the FOXP2-mNeon line could provide a tool for performing FOXP2-ChIP on mNeon-sorted FOXP2-expressing cells from cerebellar organoids to identify directly FOXP2-regulated genes. Furthermore, genetic disruption of FOXP2 expression in cerebellar organoids and observing any phenotype would enable better understanding of the specific role of FOXP2 in NDDs.

In this study, we have shown that cerebellar organoids can recapitulate cell type populations present in the developing human cerebellum, including the presence of a *FOXP2*^+^ population expressing markers of Purkinje cells and CN. Although published snRNAseq datasets from human *in vivo* samples provided a useful tool to validate our model, these resources present only static snapshots of development. In contrast, cerebellar organoids provide an *in vitro* human model to dynamically test mechanisms of cell type development and disease in the cerebellum and thus are poised to accelerate research into the many cerebellar neurodevelopmental disorders.

## MATERIALS AND METHODS

### Analysis of published datasets

Human developing brain bulk RNA-sequencing data were downloaded from BrainSpan (https://www.brainspan.org/static/download.html; accessed 6 January 2023). Processed snRNAseq data from developing human cerebella were downloaded from https://apps.kaessmannlab.org/sc-cerebellum-transcriptome/ (Sepp et al., 2024). scRNAseq data from human cerebellar organoids generated with a recently published alternative protocol (Atamian et al., 2024) were downloaded and processed following https://github.com/quadratolab/Cerebellar-Organoid-2023. The data were loaded in R, and packages SingleCellExperiment (SingleCellExperiment_1.20.0), Seurat (Seurat_5.1.0), tidyverse (tidyverse_2.0.0) and reshape2 (reshape2_1.4.4), ggplot2 (ggplot2_3.4.3) and RColorBrewer (RColorBrewer_1.1-3) were used for generating plots.

### iPSC culture

Human iPSCs were grown on Matrigel hESC-qualified matrix (Corning, 354277) and fed daily with mTeSR™1 medium (StemCell, 85850). Wells were passaged with 0.5 mM EDTA (Invitrogen, 15575020) when 70-80% confluent. Rock inhibitor (10 μM Y-27632, Abcam, ab120129) was added to mTeSR™1 medium to promote survival for the first 24 h after thawing; subsequent culture and passaging was performed without Y-27632. iPSCs were expanded to generate a working stock and frozen in freezing medium [10% DMSO (Sigma-Aldrich, D2650), 30% embryonic stem cell grade foetal calf serum (Life Technologies, 16141-079), 60% knockout Dulbecco's modified Eagle medium (Gibco, 10829)] at 2×10^6^ cells/vial. iPSCs were passaged at least once post thaw before differentiation. No antibiotics were used in iPSC culture, and cells were visibly inspected for contamination to ensure sterility. We used the control iPSC line AH017-3 (StemBANCC, STBCi026-A, ECACC 66540694, RRID:CVCL_RB85), derived from skin fibroblasts from a 67-year-old female donor by sendai virus reprogramming (Handel et al., 2016). This line has been previously characterised and used for differentiation to cerebellar organoids (Handel et al., 2016; Nayler et al., 2021). In Fig. S2C-E, we used the control iPSC line WTC11 (hPSCreg, UCSFi001-A, RRID:CVCL_Y803, CIMR), derived from skin fibroblasts of a male donor aged 30-34 years by episomal reprogramming.

### Differentiation of human cerebellar organoids

Cerebellar organoids were generated from iPSCs following an established protocol (Tolonen et al., 2024). In brief, iPSCs were dissociated using 1× TrypLE (Gibco, 12604013) and reaggregated in 96-well ultra-low attachment V-bottom plates (Greiner Bio-One, 651970) at a concentration of 1.25×10^4^ cells per well. Aggregates were seeded in induction medium [50% Iscoves MDM (Gibco, 31980022), 50% Ham's F12 (Gibco, 31765-027), 7 μg/ml insulin (Sigma-Aldrich, I3536-100MG), 5 mg/ml bovine serum albumin (BSA; Sigma-Aldrich, A4161-5G), 1× lipid concentrates (Gibco, 11905-031), 450 μM monothioglycerol (Sigma-Aldrich, M-6145), 15 μg/ml apo-transferrin (Sigma-Aldrich, T1147), 1× penicillin/streptomycin (pen/strep; Gibco, 15140122)], supplemented with 50 μM Y-27632 (Abcam, ab120129) and 10 μM SB431542 (Tocris, 1614/10). On day 2, 20 μl medium was removed and replaced with 20 μl induction medium with Y-27632 and SB431542 and 250 ng/ml FGF2 (R&D Systems, 4114-TC-01M), resulting in a concentration of 50 ng/ml FGF2 in each well. On day 7, a 1/3 volume medium change was performed using induction medium with no additional supplements. On day 14, organoids were transferred to 48-well ultra-low attachment plates (Greiner Bio-One, 677970) in 200 μl fresh induction medium per well. After 21 days, medium was switched to differentiation medium [neurobasal medium (Gibco, 21103-049), 1× GlutaMax (Gibco 35050-061), 1× N2 (Gibco 17502-048), 1× pen/strep (Gibco, 15140122)], and a complete medium change was performed every 7 days up to day 35. Beyond day 35, organoids were kept in suspension, with differentiation medium volume increased to 300 μl/well and a 1/3-1/2 volume medium change performed three times per week.

### Reverse transcription (RT)-qPCR

RNeasy Mini or Micro kits (Qiagen, 74104 or 74004) were used for isolation of RNA from iPSC pellets and cerebellar organoids. Around 20-30 organoids were sufficient for RNA extraction at day 21, 15-20 at day 35 and eight to ten at later timepoints. For each reaction, 0.5-1 μg RNA was used for generating cDNA with a SuperScript™ III First-Strand Synthesis System kit (Invitrogen, 18080051). cDNA was then diluted 1:10 for use in RT-qPCR. Primers (Table S1) were tested using a standard curve to determine efficiency, and melt curves were checked for a single qPCR product peak. RT-qPCR was performed using Fast SYBR Green Master Mix (Applied Biosystems, 4385612) on a StepOne plus Reverse transcription PCR system (Applied Biosciences). Each gene-sample pair was performed in triplicate, with the average value taken from these technical replicates. Expression of genes of interest was calculated using the dCT method relative to housekeeper genes *ACTB* and *GAPDH*. No significant changes in the expression of housekeeper genes were found across the differentiation time course. Statistical tests were performed on dCT values.

### Immunostaining

Organoids were fixed in 4% paraformaldehyde (PFA) for 15-20 min at room temperature, washed once in PBS and then transferred into 20% sucrose (Sigma-Aldrich, S9378) to equilibrate overnight at 4°C. Next, organoids were embedded in Optimal Cutting Temperature compound (Fisher Scientific, KMA-0100-00A) and frozen for cryosectioning.

Before staining, slides were warmed to room temperature and hydrated in PBS for 10 min. Sections were treated with 0.1 M glycine (Sigma-Aldrich, G8898) for 30 min, permeabilised with 0.3% Triton X-100 (Sigma-Aldrich, T9284) in PBS (PBST) for 30 min, and blocked in 2% milk (Millipore, 70166) in PBST for 1 h. Primary antibodies (Table S2) were incubated in block overnight at 4°C. After three washes with 0.05% Tween in PBS, secondary antibodies (1:1000, Alexa Fluor, Invitrogen) were incubated for 1 h. Wash steps were repeated, and nuclei were stained with 1 μg/ml Hoechst33258 (Invitrogen, H1398) in PBS before mounting in Vectashield (Vector, H-1000). Slides were imaged using a Zeiss Axioplan2 upright fluorescent microscope or a Zeiss-880 laser scanning confocal microscope.

### CRISPR-mediated generation of FOXP2-mNeon line

gRNAs targeting within 50 bp of the FOXP2 STOP codon were designed using online tools CCTop and CRISPOR. Four candidate gRNAs were selected based on high predicted specificity and efficiency scores, and a low number of off-target sites. Efficiency for each gRNA was determined by transfection with Cas9 into iPSCs as described below. A region across the FOXP2 stop codon was sanger sequenced, and the resulting traces were analysed using TIDE. The gRNA with the highest efficiency was chosen (AGAGCCTTTATCTGAAGATC).

The donor construct sequence contained sequences encoding a flexible XTEN linker (Komor et al., 2016), a P2A peptide and mNeonGreen, surrounded by two homology arms (see Supplementary Materials and Methods), and was purchased as a double-stranded DNA gBlock (IDT). ssDNA copies of the repair construct were generated using the Guide-it™ Long ssDNA Production System v2 (Takara Bio, 632666). As prediction of the relative integration efficiency of each strand is unknown, both ssDNA products were made and used in separate transfection reactions.

A ribonucleoprotein was prepared by incubating 0.5 μl Alt-R S.p. HiFi Cas9 Nuclease V3 (IDT, 20925207), 22 pmol sgRNA and 1.5 μg ssDNA in transfection enhancer buffer (Invitrogen, MPK10096) for 20 min at room temperature. For each reaction, a suspension of 2.2×10^5^ cells was mixed with the prepared ribonucleoprotein and ssDNA donor solution. Cells were electroporated using the NEON Transfection system (Invitrogen, MPK10096) and 10 μl Neon pipette tips, according to program HiTrans (1400 V, 20 ms width, one pulse), and seeded onto Matrigel-coated wells. gDNA was extracted from the resulting pools of transfected cells using a DNeasy Blood & Tissue Kit (Qiagen, 69504). To detect the desired insertion, PCR used primers outside the homology arm regions around the *FOXP2* STOP codon (Table S3), and products were run on a 1% agarose gel to visualise bands. Untransfected cells showed a clear band of ∼1.4 kb, while successful insertion generated a 2.2-kb band also present in the CRISPR pools.

To obtain stable reporter lines, clones were isolated from the pool of transfected cells. The CRISPR pool was serially diluted onto mitotically inactivated CF1 mouse embryonic feeder cells (Millipore), allowing colony formation from single cells. Colonies were picked manually using a P200 tip, selected colonies were defined, relatively small and spaced apart from others to reduce the chance of cross contamination between clones. In total, 288 colonies were picked. iPSC clones were cultured until sufficiently confluent to be passaged to replica plates, allowing freezing of cell stocks and gDNA extraction. gDNA from 245 clones was screened for successful insertion of mNeon at the *FOXP2* locus by PCR using primers spanning from outside the 5′ homology arm to inside the mNeon sequence (Table S3). Seventy-five clones produced a PCR product and were used for the next screening step. Primers spanning outside the homology arms, as described for checking the pool, were used to genotype selected clones. Seventeen clones appeared homozygous for the insertion with only a higher-molecular mass band around 2.2 kb, and 17 clones appeared heterozygous with both a 2.2-kb and wild-type 1.4-kb band. Sanger sequencing was performed on a selection of clones and verified correct sequences in two homozygous clones (A6B and B2D). These clones were expanded to create reporter line stocks to be used for following experiments, and quality control was performed on this stock.

### CRISPR clone quality control

#### Karyotype analysis

To test for gross chromosomal abnormalities, gDNA from each iPSC stock was analysed and compared to that of the parental stock. gDNA was extracted using the DNeasy Blood & Tissue Kit (Qiagen, 69504), and samples were submitted for SNP karyotyping analysis (Infinium Global Screening Array-24 v3.0, Illumina, performed by Life&Brain GmbH).

#### Mycoplasma

iPSCs were tested for mycoplasma using a MycoAlert™ PLUS Mycoplasma Detection Kit (LT07-705).

#### Off-target site sequencing

The top six highest risk off-target sites were predicted for the gRNA by CCTop (Stemmer et al., 2015) and CRISPOR (Concordet and Haeussler, 2018), and primers were designed to amplify regions of ∼200 bp around these high-risk sites (Table S3). Sanger sequencing was performed on gDNA from FOXP2-mNeon and compared to that from the parental AH017-3 stock.

#### Fluorescence-activated cell sorting (FACS) staining for pluripotency markers

iPSCs were harvested using TrypLE (Gibco, 12604013) and fixed in 2% PFA at room temperature for 10 min. Cells were then centrifuged at 400 ***g*** for 5 min before being resuspended in ice-cold methanol to permeabilise for at least 30 min at −20°C. Next, cells were washed once in FACS buffer (1% BSA in PBS) and incubated with primary antibodies against pluripotency markers TRA-1-60 and NANOG or isotope controls (Table S2) in FACS buffer at room temperature in the dark. After 1 h of staining, cells were washed twice with FACS buffer, and analysis was performed using a Cytoflex LX analyser (Beckman Coulter).

#### Trilineage differentiation

To assess the pluripotency of the iPSCs after CRISPR/Cas9 editing, a trilineage differentiation was performed according to a STEMdiff™ Trilineage Differentiation Kit (STEMCELL Technologies, 05230). After differentiation, coverslips were washed in PBS and fixed in 4% PFA for 20 min at room temperature. Fixed cells were permeabilised for 20 min using 0.4% Triton X-100 in PBS and incubated in block [5% serum (normal goat serum, Abcam, ab7481 or normal donkey serum, Abcam, ab7475), 0.1% Triton X-100 in PBS] for 1 h. Primary antibodies (Table S2) in diluent (5% serum in PBS) were applied overnight at 4°C. Following three washes in PBS, secondary antibodies were incubated for 1 h at room temperature. Three washes were performed to remove unbound antibody, with 1 μg/ml Hoechst (Invitrogen, H1398) in the final wash. Stained coverslips were imaged on a Zeiss-880 laser scanning confocal microscope.

### Western blotting

To extract protein, six to eight cerebellar organoids were lysed with manual disruption in ice-cold Pierce RIPA buffer (Thermo Fisher Scientific, 89900) supplemented with protease (cOmplete™, Roche, 04693159001) and phosphatase (PhosSTOP™, Roche, 04906837001) inhibitors. Protein samples were then diluted with NuPAGE™ LDS Sample Buffer (Invitrogen, NP007) containing 25 mM DTT and denatured by heating at 70°C for 10 min. Then, 30-50 μg of protein was loaded on a 4-12% gradient Bis-Tris gel (Invitrogen, NP0335) and run at 200 V for 50 min. Protein was then transferred to a nitrocellulose membrane over 1.5-2 h at 250 mA. The membrane was blocked with 10% milk in TBST (150 mM NaCl, 10 mM Tris-HCl pH8, 0.05% Tween 20) and primary antibodies (Table S2) incubated overnight at 4°C, diluted in 3% BSA in TBST. After washing three times in TBST, secondary antibodies (horseradish peroxidase-conjugated, GE Healthcare) were applied for 1-2 h at room temperature in 3% BSA in TBST. Washing was repeated, Pierce™ ECL Western Blotting Substrate (Thermo Fisher Scientific, 32209) was added to the membrane, and signal was captured using a Chemidoc MP imaging system.

### Live-cell imaging

A live-cell *z*-stack of a whole FOXP2-mNeon organoid was taken using a Leica Mica confocal imaging system.

### Isolation of mNeon^+^ cell population

Fifty organoids at day 63 of differentiation were washed in HHGN [1× HBSS (Invitrogen, 14180-046), 2.5 mM HEPES pH 7.3-7.5 (Invitrogen 15630-049), 35 mM glucose, 4 mM NaHCO_3_ (Sigma-Aldrich, S6297) in H_2_O] and incubated in neuronal isolation enzyme (Thermo Fisher Scientific, 88285) for 30-40 min at 37°C with resuspension every 10 min. After removal of enzyme, organoids were washed again in HHGN and gently dissociated to single cells in differentiation medium by pipetting. Dissociated cells were centrifuged at 400 ***g*** for 5 min and resuspended at 10^7^ cells/ml in FACS buffer [1% BSA (Sigma-Aldrich, A4161-5G) in PBS]. To stain dead cells, 1 μg/ml DAPI (BioLegend, 422801) was added. Cells were sorted directly into RLT lysis buffer (Qiagen, 74004) using a BD FACSAria III (BD Biosciences).

### RNA sequencing

RNA from sorted cell populations was isolated using an RNeasy Micro kit (Qiagen, 74004). RNA concentration was measured using a Quant-iT RiboGreen RNA kit (Invitrogen, R11490), and the RNA integrity number (RIN) was evaluated using High Sensitivity RNA ScreenTape (Agilent, 5067-5579) on a TapeStation. All samples submitted for sequencing had a RIN of 8.9 or above.

Library preparation and sequencing was performed by the Oxford Genomics Centre. Library preparation used 22-58 ng RNA per sample (NEBNext Ultra II Directional mRNA kit, New England BioLabs, E7760). Sequencing was performed on an Illumina NovaSeq6000 platform using 150 bp directional paired-end read sequencing. Read alignment and quantification was performed using Kallisto (Bray et al., 2016) with Ensembl release 108. Over 45 million reads were aligned for all samples (45.7-52.5 million reads, 83-87% of total reads pseudoaligned).

Transcript quantifications were imported into R and mapped to genes by tximport (tximport_1.26.1) using a txtgene object (created with makeTxDbFromEnsembl from GenomicFeatures_1.50.3). Genes with more than ten counts in four samples were included in the analysis. DESeq2 [DESeq2_1.38.2 (Love et al., 2014)] was used for differential gene expression analysis using the formula ∼Differentiation+mNeonpopulation. The lfcShrink function type ‘apeglm’ was applied to remove noise from low-expressed genes (Zhu et al., 2019). Genes with *P*adj (Benjamini–Hochberg method to correct for multiple testing) below 0.05 were determined to be significantly differentially expressed. For visualising, gene expression was normalised using variance-stabilising transformation (VST); additional plots were made using ggplot2 (ggplot2_3.4.0) and Pheatmap (pheatmap_1.0.12).

GSEA was performed on VST-normalised expression output from DESeq2 through the online tool (GSEA 20.4.0 on the GenePattern server) (Mootha et al., 2003; Subramanian et al., 2005). All samples and genes above the minimum counts threshold described above were included in the GSEA. Analysis used 1000 repetitions, permutated on gene sets, and a FDR threshold of 0.05 for significant terms. GO terms were used from the MSigDB on the GenePattern server, and cerebellar markers and disorder gene sets were uploaded. Gene lists for cerebellar markers and disorders are found in Tables S4-S9 and were determined as differentially expressed genes (absolute log2FC 1.5, *P*adj<0.05) from laser-capture micro-dissected fetal human cerebellar samples (Aldinger et al., 2021). Cerebellar cell type marker genes were extracted using scran::FindMarkers() from developing human snRNAseq (Sepp et al., 2024). Disorder gene lists were previously used for analysis of human cerebellar data (Aldinger et al., 2021), with addition of ASD genes categorised as class 1 (high confidence) by Simons Foundation Autism Research Initiative (SFARI; 23 January 2023 release) and CAS genes compiled from publications that performed whole-genome sequencing of probands with CAS and close family members (Eising et al., 2019; Hildebrand et al., 2020; Kaspi et al., 2022). Genes differentially expressed in autopsy brain samples (frontal and temporal cerebral cortex) of individuals with different neurological disorders were published by Gandal et al. (2018).

When comparing overlap with FOXP2 target genes (Hickey et al., 2019; Spiteri et al., 2007; Vernes et al., 2007), the criteria for differentially expressed genes between mNeon-sorted organoid populations was an *P*adj<0.05 and absolute log2FC>1.5. GeneOverlap (GeneOverlap_1.34.0) was used to perform a Fisher's exact test to determine the significance of overlap.

### Quantification and statistical analysis

Statistical tests were performed in R. Significant differences between two groups were determined using Student’s *t*-test. For more than two groups, ANOVA followed by post-hoc Tukey's HSD test was used to identify significant differences. Unless overwise stated, *P*<0.05 was considered significant. Details of statistical tests are found in results and figure legends. Each biological replicate (*n*) resulted from one or more organoid(s) from an independent differentiation batch; therefore, each experiment used three or more differentiations.

## Supplementary Material

10.1242/dmm.052290_sup1 Supplementary information

Table S4. Cerebellar regions (related to Fig. 4C).

Table S5. Cerebellar cell types (related to Fig. 4G).

Table S6. FOXP2-ChIP targets significantly differentially expressed in mNEON-sorted cerebellar organoid RNAseq (related to Fig. 5C).

Table S7. Overlap between genes differentially expressed in presence/absence of FOXP2 with cerebellar organoid data (related to Fig. 5D).

Table S8. GSEA gene set input (related to Fig. 5E).

Table S9. GSEA gene set input (related to Fig. 5H).
