# Supplementary material for: Cerebellar organoids model cell type-specific FOXP2 expression during human cerebellar development
Source: Dis Model Mech. 2025 Nov 14;18(11):dmm052290. doi: 10.1242/dmm.052290 (PMC12661646; doi:10.1242/dmm.052290)
Supplement: Supplementary information [file dmm-18-052290-s1.pdf]

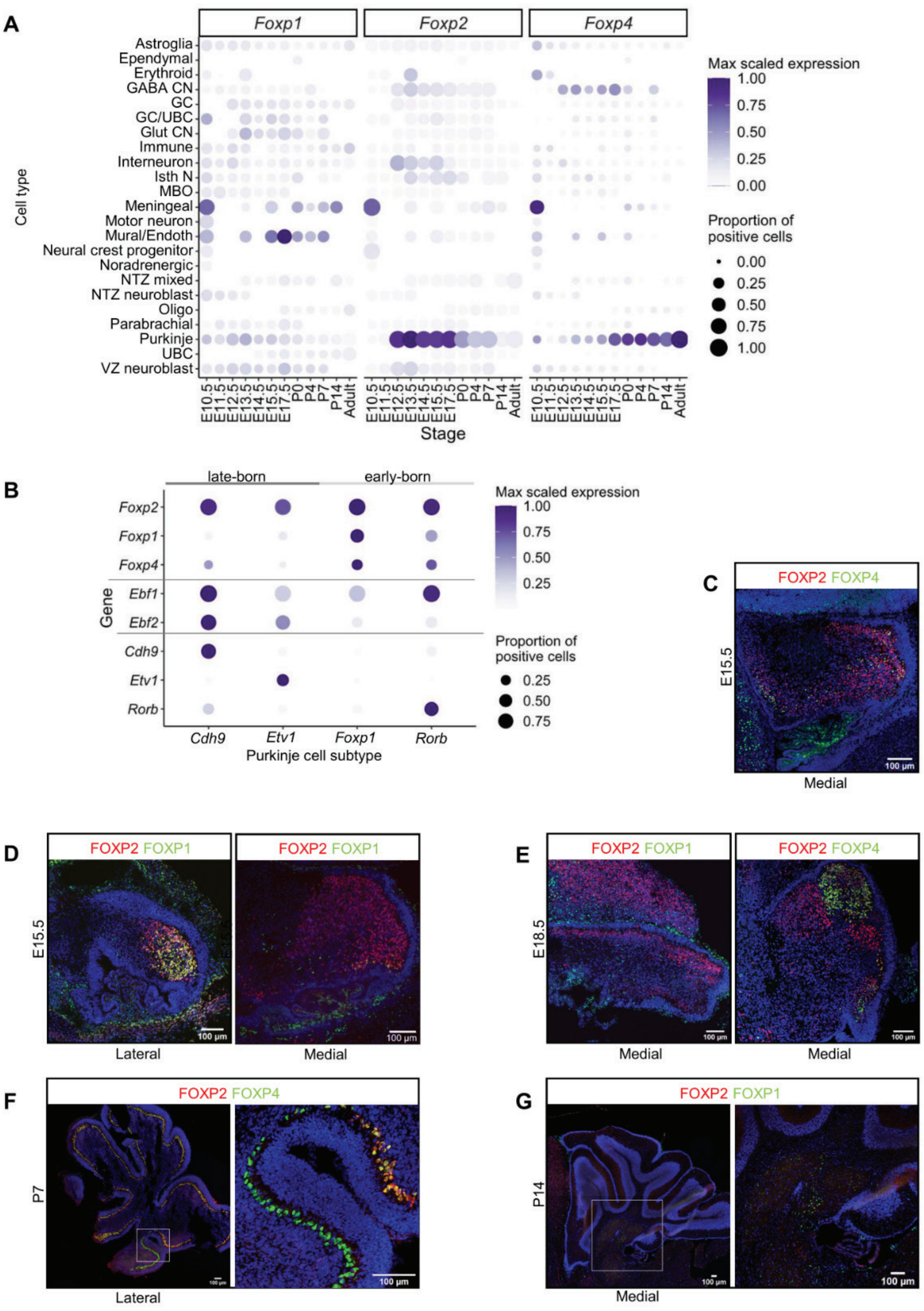

Fig. S1. *Foxp* family genes are expressed in developing mouse Purkinje cells.
(A) Expression of *Foxp* genes in the developing mouse cerebellum, plotted across timepoints and cell types based on published snRNA-seq data from Sepp et al., 2024. Point size indicates the proportion of expressing cells in each cluster and the colour scale shows average

Disease Models & Mechanisms • Supplementary information

expression levels. Samples with <10 cells were excluded. (B) *Foxp* genes show different expression patterns across mouse embryonic Purkinje cell subtypes. Purkinje cell subtypes can be distinguished by levels of *Ebf1* and *Ebf2*, and high expression of markers *Cdh9*, *Etv1*, *Foxp1* and *Rorb*. Analysis based on snRNAseq data from Sepp et al., 2024. **(C-G)** Immmunostaining for FOXP proteins FOXP2 (red) with FOXP1 or FOXP4 (green) across mouse cerebellar development confirm expression in developing Purkinje cells. Images from E15.5 (C, D), E18.5 (E), P7 (F) and P14 (G). FOXP1 and FOXP4 display differential expression across Purkinje cell clusters. In addition, FOXP1 and FOXP2 show sparse expression in the white matter of post-natal samples. Nuclei labelled by Hoechst (blue), scale bars = 100 µm. Insert locations indicated by white box in E&F.

Disease Models & Mechanisms • Supplementary information

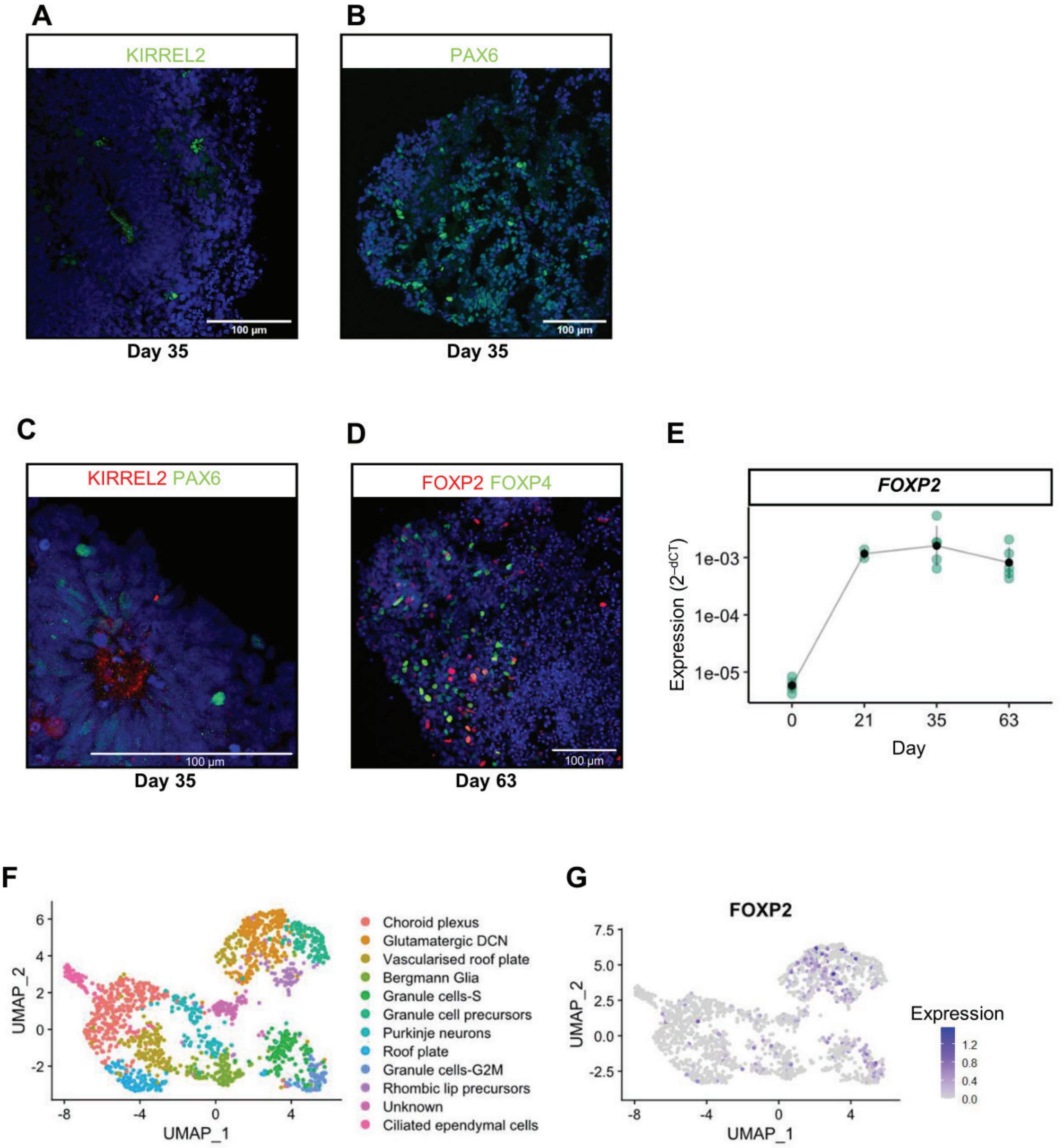

Fig. S2. Cerebellar organoids generate cells from both germinal zones.
Immunostaining of day 35 cerebellar organoids generated from AH017-3 iPSC line using antibodies against **(A)** KIRREL2 labelling the ventricular zone and **(B)** PAX6 labelling the rhombic lip lineage. Nuclei labelled by Hoechst (blue), scale bars = 100 µm. **(C)** Immunostaining of day 35 cerebellar organoids generated from WTC11 iPSC line using antibodies against KIRREL2 (red) and PAX6 (green). Nuclei labelled by Hoechst (blue), scale bars = 100 µm. **(D)** Immunostaining of day 63 WTC11 cerebellar organoids with antibodies against FOXP2 (red) and FOXP4 (green). Nuclei labelled by Hoechst (blue), scale bars = 100 µm. **(E)** Expression of *FOXP2* measured by RT-qPCR in cerebellar organoids generated using WTC11 iPSCs. Expression calculated as 2^{-dCT}, relative to housekeeper genes *ACTB* and *GAPDH*. Green points represent individual differentiations (n=4), black points show mean and error bars representing mean±SD. **(F)** Cerebellar cell types identified in day 90 cerebellar organoids. scRNAseq from Nayler *et al.,* 2021. **(G)** Expression of *FOXP2* in day 90 cerebellar organoids, scRNAseq from Nayler *et al.,* 2021.

Disease Models & Mechanisms • Supplementary information

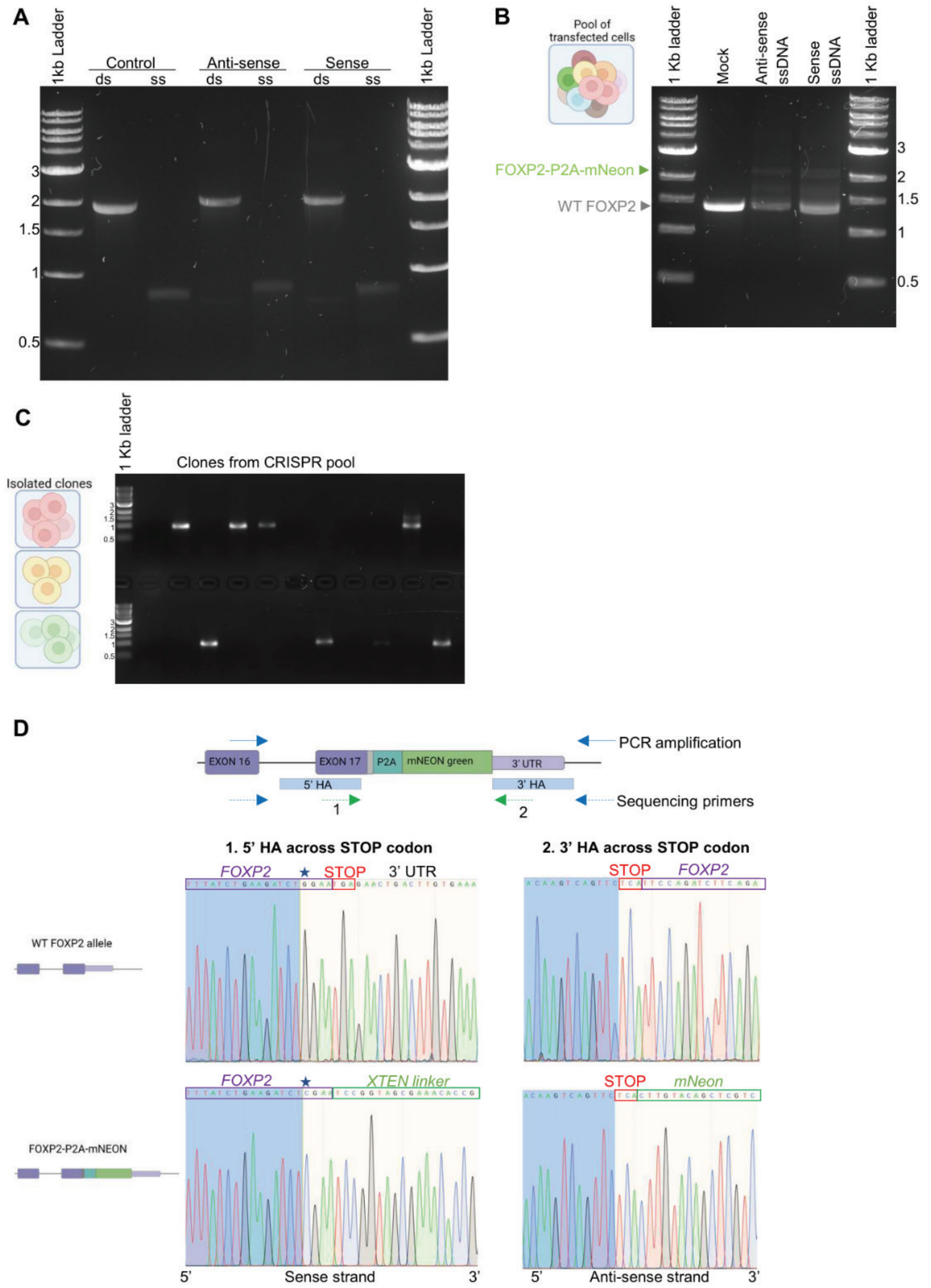

Fig. S3. CRISPR/Cas9-mediated generation of FOXP2-mNeon reporter line.
(A) The size and purity of the ssDNA donor construct was checked before transfection. ssDNA products run at approximately half the size of the parental dsDNA (2.1 kilobase pairs (kb)) and show clean bands indicating full digestion. **(B)** Pools of transfected cells show insertion of P2A-mNeon when tested by PCR and visualised on a 1% agarose gel. From untransfected cells, the PCR generated a wild-type (WT) band ~1.4 kb. Transfections with either ssDNA donor resulted in some cells with successful insertion detected by an additional band at ~2.2 kb, with similar efficiency. **(C)** To screen clones for insertion, a primer pair from outside the 5' homology region to within the mNeon sequence were used. PCR products were visualised on a 1% agarose gel, with the presence of a band showing mNeon insertion. **(D)** Sanger sequencing of the genotyping PCR product in (C) confirmed the accurate insertion of the mNeon.

Disease Models & Mechanisms • Supplementary information

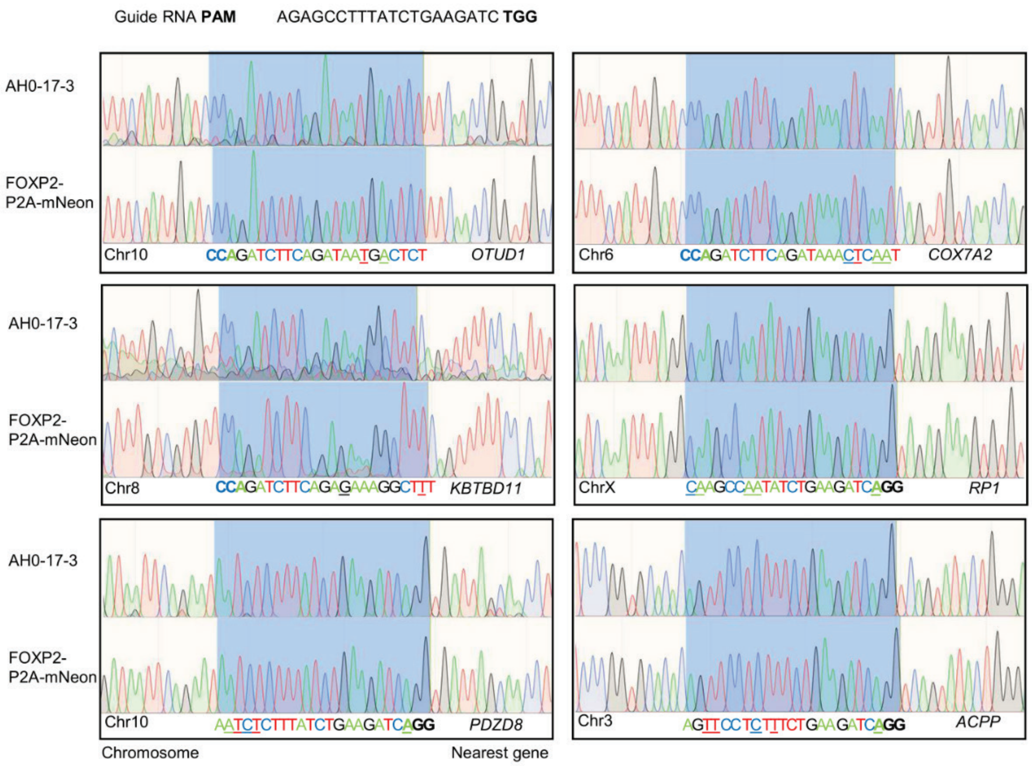

Fig. S4. The sequences of predicted off-target sites were not altered in the FOXP2-mNeon line.

The predicted top six off-target sites for the gRNA used for FOXP2 editing were sequenced to check for mutations in the FOXP2-mNeon line. Sequencing traces are shown for the parental AH017-3 line and FOXP2-mNeon reporter line clone B2D. Sites with a close match to the gRNA target sequence and their adjacent PAM motif are highlighted in blue. Sequences are shown below with mismatches to gRNA target site underlined and the PAM sequence in bold. The off-target loci are labelled with their chromosome and nearest gene.

Disease Models & Mechanisms • Supplementary information

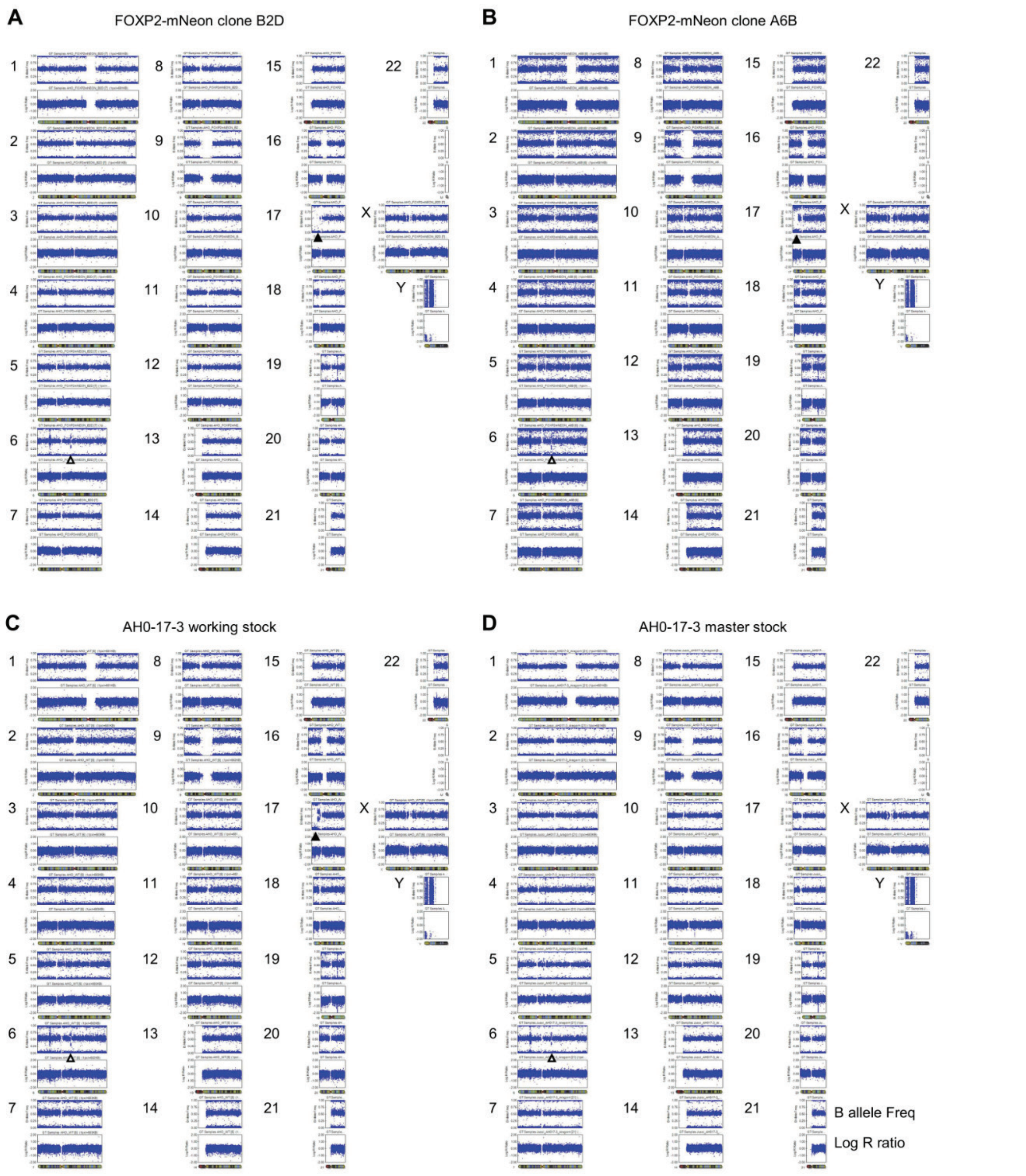

Fig. S5. Karyograms from SNP analysis of FOXP2-mNeon clones and parental iPSC line.

SNP array analysis on genomic DNA was used to assemble karyograms for the iPSC stocks. Karyotypes are shown for two FOXP2-mNEON reporter line clones **(A)** B2D & **(B)** A6B, **(C)** the parental AH017-3 working stock used to generate this line and **(D)** an alternative AH017-3 master stock. The control iPSC line, AH017-3, had a small duplication when established (Chr 6q14.1 2mb duplication) indicated by the open triangle in all 4 samples (Handel *et al.,* 2016). However, the AH017-3 stock used for reporter line generation shows an additional chromosomal alteration on chromosome 17 which was consequently shared in both FOXP2-P2A-mNeon clones. The solid triangle indicates the Chr17p13-12 13mb loss of heterozygosity (LOH) and Chr17p12-11 6mb duplication.

Disease Models & Mechanisms • Supplementary information

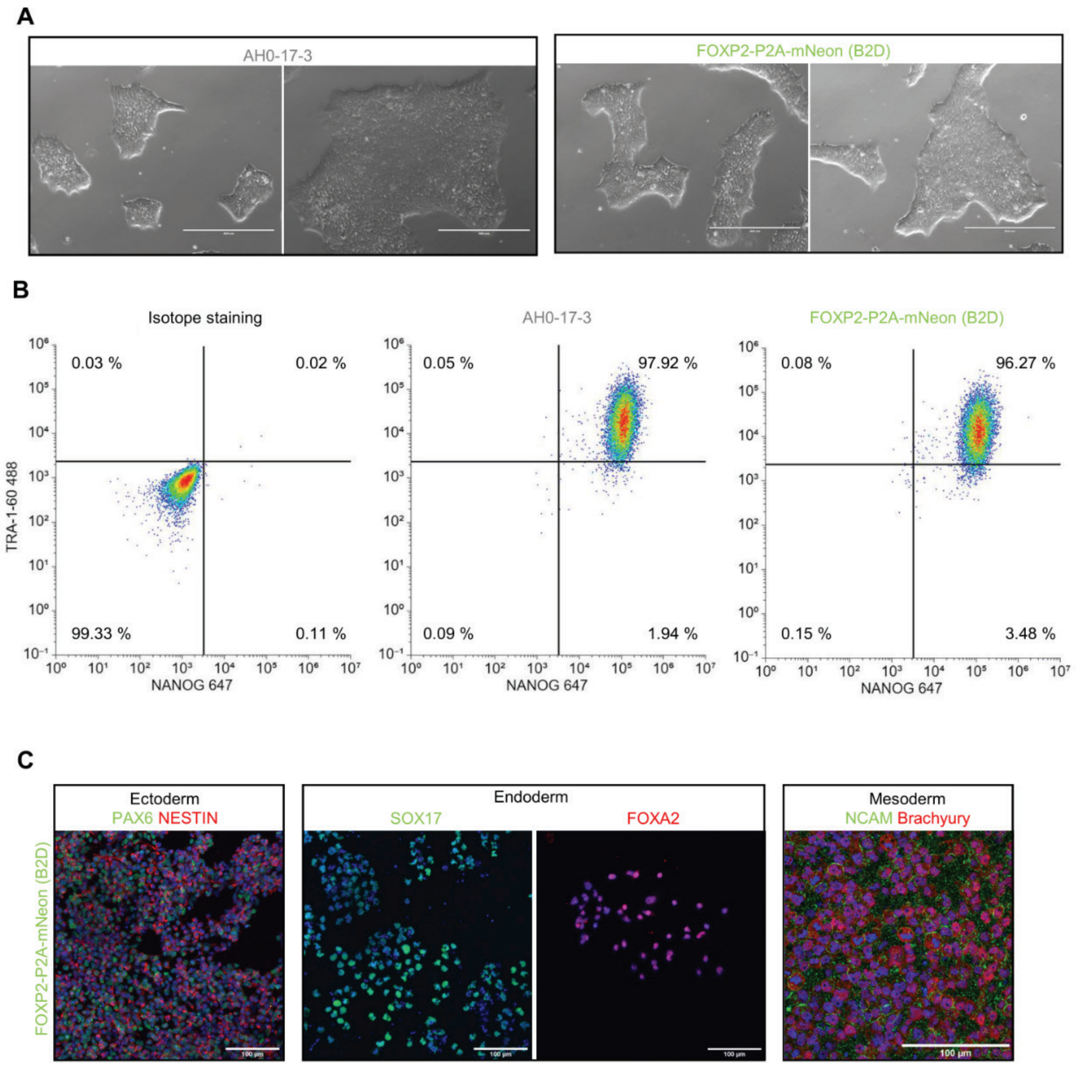

Fig. S6. FOXP2-mNeon line clone B2D displays pluripotent state.

(A) Brightfield images of control parental line (AH017-3) and FOXP2-mNeon reporter line (clone B2D) showing iPSC colonies with bright borders and tightly packed cells with large nuclei. Scale bars = 400 µm. **(B)** Flow cytometry analysis was used to quantify expression of pluripotency markers. iPSCs were stained with fluorescent-conjugated antibodies for TRA-1-60^{488} and $NANOG^{647}$ or with isotype controls IgM^{488} and IgG^{647} to set gating for positive test antibody staining. The population percentages for each quadrant are indicated. **(C)** To confirm the capability to differentiate into all three germ lineages, a trilineage differentiation was performed from FOXP2-mNeon iPSCs (clone B2D). Differentiated cells were fixed and stained using antibodies against markers for each lineage. Ectoderm cells were stained with antibodies against PAX6 (green) and NESTIN (red), endoderm cells separately with SOX17 (green) and FOXA2 (red), and mesoderm cells with NCAM (green) and Brachyury (red). Nuclei stained with Hoechst (blue), scale bars = 100 µm.

Disease Models & Mechanisms • Supplementary information

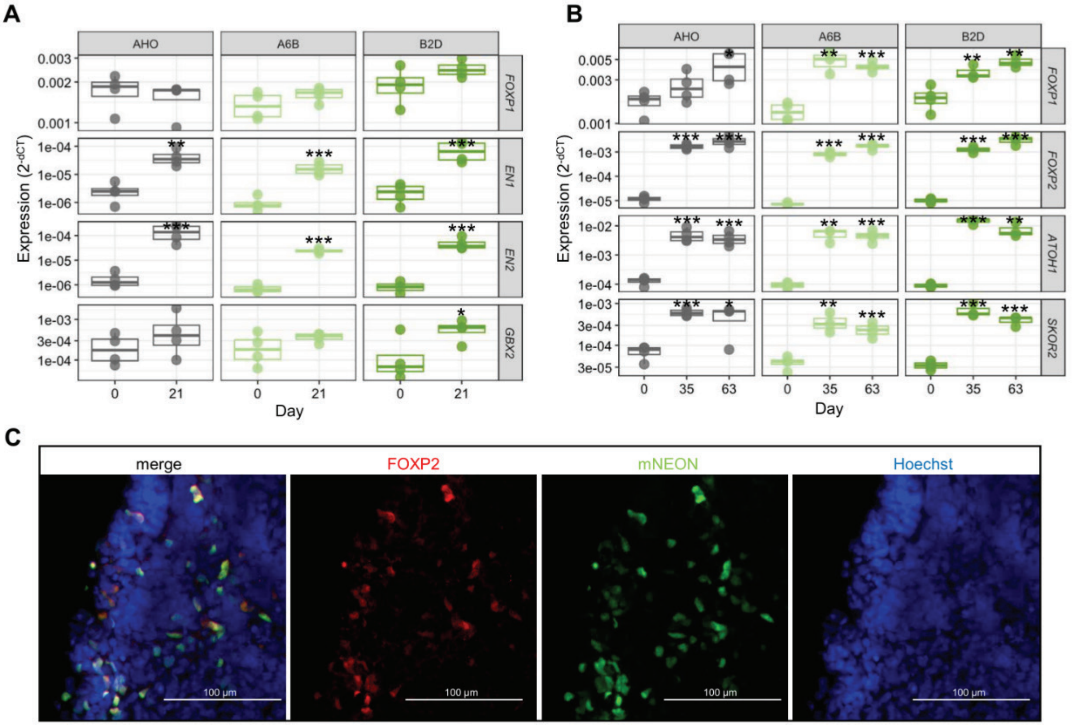

Fig. S7. Characterisation of FOXP2-mNeon reporter line cerebellar organoids.
Upregulation in expression of marker genes for early cerebellar specification **(A)** and cerebellar lineages **(B)** across cerebellar organoid differentiation in parental AH017-3 and two FOXP2-mNeon reporter line clones (A6B & B2D). Data replotted from Figure 3D. Expression calculated as 2^{-dCT}, relative to housekeeper genes *ACTB* and *GAPDH*. Pale points represent individual differentiations (n=4). Significant change in expression compared to day 0 determined using a t-test on dCT values. *$p<0.05$, **$p<0.01$, ***$p<0.001$. **(C)** Immunofluorescent staining of FOXP2 (red) and mNeon (green) confirms co-expression in sections of a day 63 cerebellar organoid generated using FOXP2-mNeon clone A6B. Nuclei were stained with Hoechst (blue). Scale bars = 100 µm.

Disease Models & Mechanisms • Supplementary information

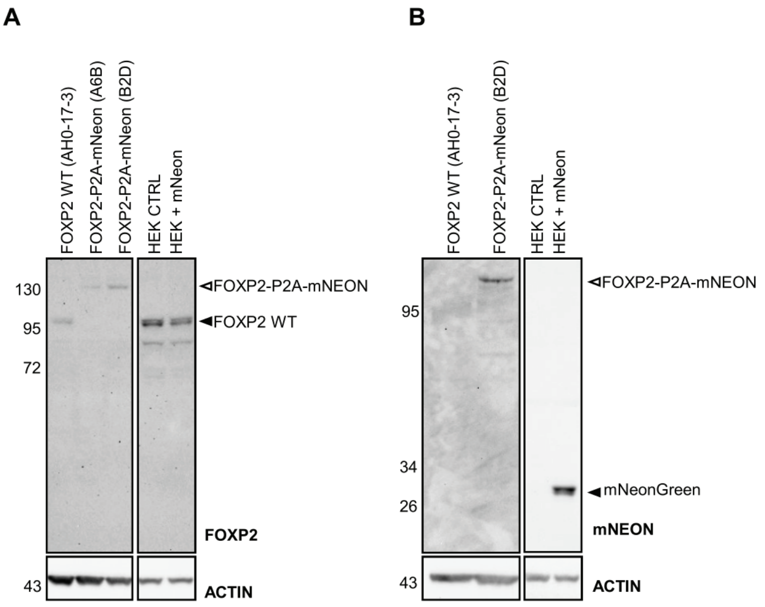

Fig. S8. FOXP2-mNeon line shows incomplete cleavage of FOXP2 and mNEON.
(A) Western blotting for FOXP2 shows a higher molecular weight band in two FOXP2-mNeon reporter line clones (A6B & B2D) at ~130 kDa, the predicted size of a FOXP2-mNeon fusion protein. A wild-type (WT) band for FOXP2 at around 95 kDa is present in the WT (AH017-3) cerebellar organoid and HEK293 cell samples. Protein samples were lysed from day 63 cerebellar organoids. ACTIN is shown as a loading control. **(B)** Western blotting for mNeonGreen shows a high molecular weight band in two FOXP2-mNeon reporter line clones (A6B & B2D) at ~ 130 kDa, the predicted size of a FOXP2-mNeon fusion protein. A band at 26kDa is detected in HEK293 cells with exogenous expression of mNeon. Samples are shown from HEK293 cells that were untransfected (HEK CTRL) or transfected with a plasmid to express mNeongreen (HEK+mNEON). ACTIN is shown as a loading control.

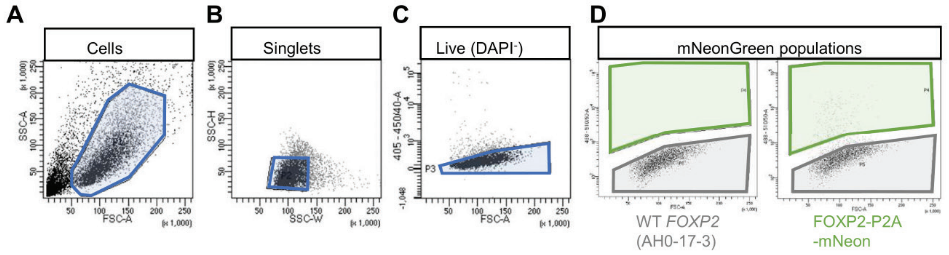

Fig. S9. Isolation of mNeon positive and negative cells, by flow cytometry.
(A) Cells were selected from debris and other particles using a plot of forward scatter (x axis) against side scatter (y axis). **(B)** Single cells were then selected based on side scatter height (x axis) and side scatter width (y axis). **(C)** Live cells were selected based on low signal for DNA binding dye DAPI indicating intact membranes. Fluorescence intensity from 405 laser excitation on the y axis. **(D)** Finally, mNeon positive and negative populations were selected according to fluorescent intensity (y axis). Gating thresholds were determined using WT cerebellar organoids (left) which had no mNeon expression.

Disease Models & Mechanisms • Supplementary information

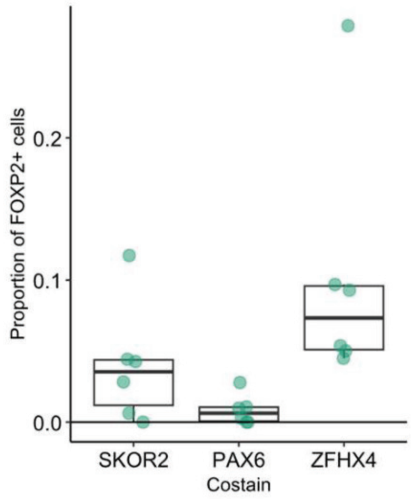

Fig. S10. Quantification of FOXP2 co-expression with markers of cerebellar cell types.

The proportion of FOXP2-positive cells also positive for indicated marker SKOR2 (Purkinje cells), PAX6 (granule cells), and ZFHX4 (cerebellar nuclei neurons). Data points represent separate differentiations (n=5), calculated from imaging of at least four organoid sections per differentiation.

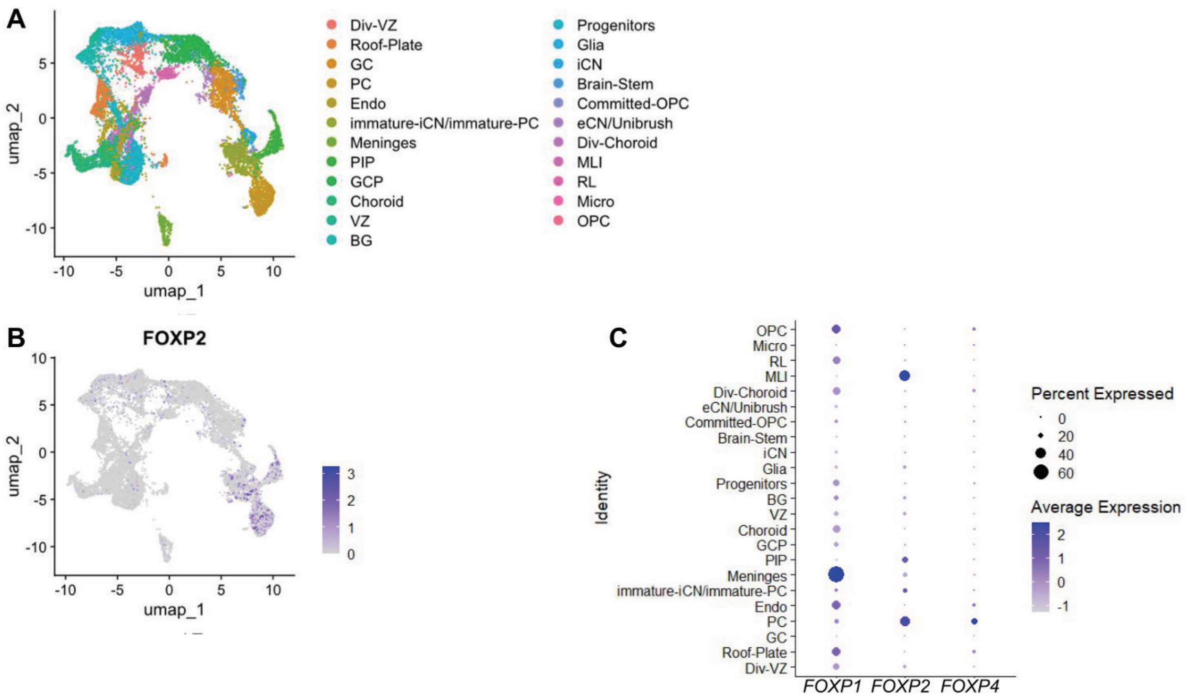

Fig. S11. *FOXP2* expression in cerebellar organoids generated with alternative protocol.

(A) Cerebellar cell types identified in cerebellar organoids generated by Atamian *et al.,* 2024. **(B)** FOXP2 expression in Atamian *et al.,* 2024 cerebellar organoids. **(C)** Expression of FOXP family genes across cell types in Atamian *et al.,* 2024 cerebellar organoids. Point size indicates the proportion of expressing cells in each cluster and the colour scale shows average expression levels. OPCs, oligodendrocyte progenitor cells; RL, rhombic lip; MLIs, molecular layer interneurons; Div−, dividing; eCNs/Unibrush, excitatory cerebellar nuclei and unipolar brush cells; iCNs, inhibitory cerebellar nuclei; BG, Bergmann glia; VZ, ventricular zone; GCP, granule cell progenitors; PIPs, Pax2+ interneuron progenitors; PCs, Purkinje cells; GC, granule cells.

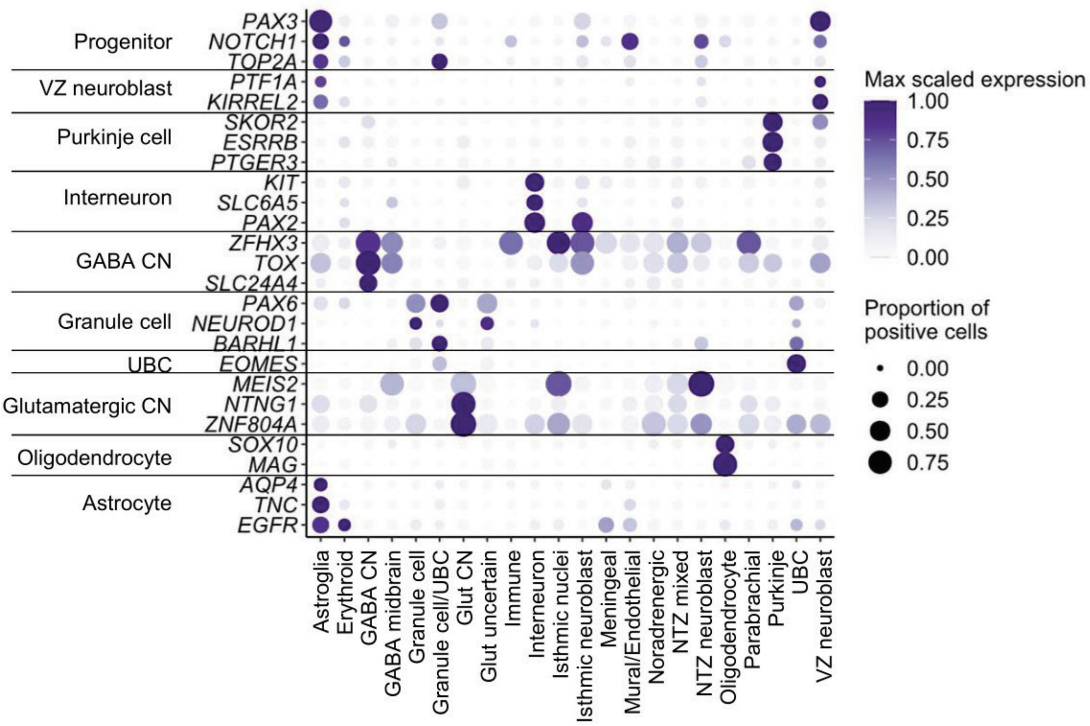

Fig. S12. Expression of selected cerebellar cell type markers show specificity in human snRNAseq.

Expression of selected cerebellar cell types markers in published developing human snRNAseq (Sepp et al., 2024). Point size indicates the proportion of expressing cells in each cluster, and the colour scale shows average expression levels. Samples with <10 cells were excluded. CN, cerebellar nuclei neurons; glut: glutamatergic; NTZ; nuclear transitory zone; UBC: unipolar brush cells; VZ: ventricular zone.

Disease Models & Mechanisms • Supplementary information

Table S1. RT-qPCR primers

Target	Fw primer	Rv primer
ACTIN	GCCGCCAGCTCACCATGGATG	CCATCACGCCCTGGTGCCTGG
ATOH1	TGTTATCCCGTCGTTCAACAAG	TGGGCGTTTGTAGCAGCTC
EN1	GCTTGTCCTCCTTCTCGTTC	TGGTCAAAACTGACTCGCAG
EN2	CCGGCGTGGGTCTACTGTA	GGCCGCTTGTCCTCTTTGTT
FOXP1	CTTCAGCTTCCTCTGGATCG	GGCAGATCTCCTATGCAAGC
FOXP2	TGGCAGCAGAGATGGAAGAT	AGTTGTCTTGCTGCCTGGAG
FOXP4	AATGGTGAGATGAGTCCCGC	TTTGCTGTCATTGTTCCCTGG
GAPDH	GGAAGGTGAAGGTCGGAGTC	GTTGAGGTCAATGAAGGGGTC
GBX2	AAAGAGGGCTCGCTGCTC	GGTCGTCTTCCACCTTTGAC
SKOR2	AGCCCAGTTCACCATCCAT	GCTGTTGTCATCCTTTGTAGATAC

Table S2. Primary antibodies

Target	Supplier	Code	Source	Use	Concentration
Actin	Abcam	Ab179467	Rabbit	WB	1:1000
Brachyury	R&D systems	AF2085	Goat	IF	1:100
CALB1	Swant	CB38	Rabbit	IF	1:10,000
FOXA2	R&D systems	AF2400	Goat	IF	1:200
FOXP1	CST	2005	Rabbit	IF	1:500
FOXP2	Abcam	Ab1307	Goat	IF	1:1000
FOXP2	Sigma	MABE415	Mouse	WB	1:1000
FOXP4	Atlas	HPA007176	Rabbit	IF	1:1000
GBX2	ThermoFisher	H00002637-M01	Mouse	IF	1:100
IgG 647	Cell Signalling	2985S	Rabbit	FACS	5 µg/ml
IgM 488	Biolegend	401617	Mouse	FACS	15 µg/ml
KIRREL2	R&D	Af2930	Goat	IF	1:250
MAP2	Abcam	Ab183830	Rabbit	IF	1:500
mNeonGreen	Chromotek	32F6	Mouse	IF	1:1000
mNeonGreen	Chromotek	4M2	Rabbit	WB	1:1000
NANOG 647	Cell Signalling	5448S	Rabbit	FACS	5 µg/ml
NCAM	Abcam	ab75813	Rabbit	IF	1:200
NESTIN	Sigma	MAB5326	Mouse	IF	1:500
PAX6	GeneTex	GTX113241	Rabbit	IF	1:500
SKOR2	Atlas antibodies	HPA046206	Rabbit	IF	1:500
SOX17	R&D systems	AF1924	Goat	IF	1:100
TRA-1-60 488	Biolegend	330614	Mouse	FACS	15 µg/ml
ZFHX4	Atlas antibodies	HPA023837	Rabbit	IF	1:500

Table S3. PCR primers used in FOXP2-mNeon screening and quality control

Purpose	Name	Fw primer	Rv primer	Product size (bp)
Sequencing *FOXP2* homology arms Clone screening: genotyping	FOXP2_HAseq	TTGAAAATAGTACTGGCATCCTGG	CTATGGTGCTTGCACAGATAATG	WT *FOXP2*: 1438 FOXP2-P2A-mNeon: 2245
Additional *FOXP2* sequencing	FOXP2_HAs_midse2	GCCAGTGATTGCAGAGGATG	–	NA
Additional *FOXP2* sequencing	FOXP2_HAseq_midseq_rv	–	TTTGGCAGTATATGAGGCGC	NA
FOXP2 gRNA testing	FOXP2_gRNA_test	CAATCCACGTCAAGGAAGAGC	TGCCTGTTGGTACTGAATCC	314
Generation of P2A-mNeon ssDNA repair construct	FOXP2_ssDNA	TTCAACAAACACCCAATCTAGC	CAGCACAGACTGGAAGCCTA	2221
Clone screening insertion	FOXP2mNEON_screen	TGGGGTTGGAGGGATAATAAACTT	CCATCATTTGGATTGCCGGT	WT *FOXP2*: no product *FOXP2-P2A-mNeon*: 1081
Off-target testing	10_OTUD1	AAGAGCTCTGAAGCCTGCTG	AAGTGTGGGGATTACAGGCG	196
Off-target testing	8_KBTBD11	GCACCATTGTACTCCAGCCT	AGCGGAACACAATAAATGTGGA	133
Off-target testing	10_PDZD8	GCGTTACTGTTGTGGGAGGA	GCCCTTAGAGCTGTCTGCAT	231
Off-target testing	6_COX7A2	GTCCGCTGAGAGTTTCCTCC	TTTGGTTCAGAAAGGCGGGA	506
Off-target testing	X_RP1	GAGTACACCCAAGGCCTGATT	ACACATATACCTTCCCAACACACT	188
Off-target testing	3_ACPP	ACGTTGACCGGACTTTGATG	ACAACTGTTGAGTAGCCTGC	206

Table S4. Cerebellar regions (related to Fig. 4C).

Available for download at
https://journals.biologists.com/dmm/article-lookup/doi/10.1242/dmm.052290#supplementary-data

Table S5. Cerebellar cell types (related to Fig. 4G).

Available for download at
https://journals.biologists.com/dmm/article-lookup/doi/10.1242/dmm.052290#supplementary-data

Disease Models & Mechanisms • Supplementary information

Table S6. FOXP2-ChIP targets significantly differentially expressed in mNEON-sorted cerebellar organoid RNAseq (related to Fig. 5C).

Available for download at
https://journals.biologists.com/dmm/article-lookup/doi/10.1242/dmm.052290#supplementary-data

Table S7. Overlap between genes differentially expressed in presence/ absence of FOXP2 with cerebellar organoid data (related to Fig. 5D).

Available for download at
https://journals.biologists.com/dmm/article-lookup/doi/10.1242/dmm.052290#supplementary-data

Table S8. GSEA gene set input (related to Fig. 5E).

Available for download at
https://journals.biologists.com/dmm/article-lookup/doi/10.1242/dmm.052290#supplementary-data

Table S9. GSEA gene set input (related to Fig. 5H).

Available for download at
https://journals.biologists.com/dmm/article-lookup/doi/10.1242/dmm.052290#supplementary-data

Supplementary Materials and Methods

Analysis of mouse cerebellar samples

Wildtype mice (C57BL/6JOlaHsd) were sacrificed, and cerebella extracted under Home Office Project License PP4924146. Cerebellar samples were fixed in 4% PFA overnight at 4ºC and then cryoprotected in 30% sucrose. Immunostaining was performed as for organoid samples.

Repair construct sequence for FOXP2-mNeon generation

```
GTATTTGTTTCAACAAACACCCAATCTAGCTTATACTGGAGAGTTTAAAAAAAAATAGAAATAAAATT
GTTAAACTCAAGTCTGAAATATTTAGAAATAACAGTTGTTATCTTAATTGCTAGCAAAAAACTGAATG
GTAAAAGTGTTTTCATTTGTTGATTAATTATTTGTTTACTATTCCTTTACTACCACTCCTTGATTATC
ATGTTATAACAAATACTAACAAAACATTTCATTCAGAAAGAATGCTGAGAAACGGATGTTTTACCTAA
ACAGCACAGCATGTGGGAGTCCATGCTCACATTACTCTGAATCCCTGCCTCCACAGTTCTACTGTAAA
GTATCATGTCTCTAACCAGCTCACGCAATCAATCAGATGCCCTTTTAAGAAAGACAATGTTGTGTCTT
CTTGCCTTTTTTCAGTTGACCTCTTCACTGCAAAGTTGGCCAAACTCTGTTTGTTGCTTACTTAGTAA
AATTTTGGTGTATCTACATGTTTTTCAGACATTCAATCCACGTCAAGGAAGAGCCAGTGATTGCAGAG
GATGAAGACTGCCCAATGTCCTTAGTGACAACAGCTAATCACAGTCCAGAATTAGAAGACGACAGAGA
GATTGAAGAAGAGCCTTTATCTGAAGATCTCGAATCCGGTAGCGAAACACCGGGGACTTCAGAATCGG
CCACCCCGGAGTCTGCCACGAACTTCTCTCTGTTAAAGCAAGCAGGAGACGTGGAAGAAAACCCCGGT
CCTATGGTGAGCAAGGGCGAGGAGGATAACATGGCCTCTCTCCCAGCGACACATGAGTTACACATCTT
TGGCTCCATCAACGGTGTGGACTTTGACATGGTGGGTCAGGGCACCGGCAATCCAAATGATGGTTATG
AGGAGTTAAACCTGAAGTCCACCAAGGGTGACCTCCAGTTCTCCCCCTGGATTCTGGTCCCTCATATC
GGGTATGGCTTCCATCAGTACCTGCCCTACCCTGACGGGATGTCGCCTTTCCAGGCCGCCATGGTAGA
TGGCTCCGGATACCAAGTCCATCGCACAATGCAGTTTGAAGATGGTGCCTCCCTTACTGTTAACTACC
GCTACACCTACGAGGGAAGCCACATCAAAGGAGAGGCCCAGGTGAAGGGGACTGGTTTCCCTGCTGAC
GGTCCTGTGATGACCAACTCGCTGACCGCTGCGGACTGGTGCAGGTCGAAGAAGACTTACCCCAACGA
CAAAACCATCATCAGTACCTTTAAGTGGAGTTACACCACTGGAAATGGCAAGCGCTACCGGAGCACTG
CGCGGACCACCTACACCTTTGCCAAGCCAATGGCGGCTAACTATCTGAAGAACCAGCCGATGTACGTG
TTCCGTAAGACGGAGCTCAAGCACTCCAAGACCGAGCTCAACTTCAAGGAGTGGCAAAAGGCCTTTAC
CGATGTGATGGGCATGGACGAGCTGTACAAGTGAGAACTGACTTGTGAAACGTCAGCGTGAAGGGACA
TATCACTGACCTTCATAACCACTCCACAACCATGAATATTTGACAAATTTTTACTGTGACTATTTATT
AAGCATGGATAAAGGAGACAGCCCTAAAGGAACTTACTAAGCCAGCCCTTTGGGATTCAGTACCAACA
GGCAAATTGCTTGTTTTCTTCTTCTTCTTCTTTTTTTTTTTTTAGAAAAAAAAGACAAAAACTGATTT
TCTTGAAAAAAAAAAATGAACTGTTCTTTCTATAATGGCTTTGCCCATTTAAAAAATGTGGCTCTTAA
GGGTTCATGAAATGACTGAATATGAGGATACATGTCCTGTAGAAAGCAAATGCGCCTCATATACTGCC
AAAAATAGTGTTAGTTTCATTAATGTGAATTTTCCAGCATTCAGTAGTTGTAATGTTAGAAACAATTG
CTGGTCAAGTTCAACTTGTTGCTATTGTTTTTAATTTGCACAGGAGTAGTATCAGAAATTAGTGTCAC
TGCTTGTATCTAGCTGAATTTTAAACAACAGAACATTAGTTTTTTATGTTGGTGCCACCAACTGTAAA
TGACATAAGTTAGTTATTACAAAACACAGTAATTAGACTGTTGCAACCATCTAAAACCTTAGGCTTCC
AGTCTGTGCTG
```
